# Association mapping in sunflower (Helianthus annuus L.) reveals independent control of apical vs. basal branching

**DOI:** 10.1186/s12870-015-0458-9

**Published:** 2015-03-11

**Authors:** Savithri U Nambeesan, Jennifer R Mandel, John E Bowers, Laura F Marek, Daniel Ebert, Jonathan Corbi, Loren H Rieseberg, Steven J Knapp, John M Burke

**Affiliations:** Department of Plant Biology, Miller Plant Sciences, University of Georgia, Athens, GA 30602 USA; Present address: Department of Horticulture, University of Georgia, Athens, GA 30602 USA; Present address: Department of Biological Sciences, University of Memphis, Memphis, TN 38152 USA; North Central Regional Plant Introduction Station, Iowa State University/USDA-ARS, Ames, IA 50014 USA; Department of Botany, University of British Columbia, Vancouver, BC V6T 1Z4 Canada; Present address: Department of Crop and Soil Sciences, University of Georgia, Athens, GA 30602 USA; Department of Plant Sciences, University of California, Davis, CA 95616 USA

**Keywords:** Apical dominance, Association mapping, Branching, *Helianthus annuus*, Linkage disequilibrium, Plant architecture, Sunflower

## Abstract

**Background:**

Shoot branching is an important determinant of plant architecture and influences various aspects of growth and development. Selection on branching has also played an important role in the domestication of crop plants, including sunflower (*Helianthus annuus* L.). Here, we describe an investigation of the genetic basis of variation in branching in sunflower via association mapping in a diverse collection of cultivated sunflower lines.

**Results:**

Detailed phenotypic analyses revealed extensive variation in the extent and type of branching within the focal population. After correcting for population structure and kinship, association analyses were performed using a genome-wide collection of SNPs to identify genomic regions that influence a variety of branching-related traits. This work resulted in the identification of multiple previously unidentified genomic regions that contribute to variation in branching. Genomic regions that were associated with apical and mid-apical branching were generally distinct from those associated with basal and mid-basal branching. Homologs of known branching genes from other study systems (i.e., *Arabidopsis*, rice, pea, and petunia) were also identified from the draft assembly of the sunflower genome and their map positions were compared to those of associations identified herein. Numerous candidate branching genes were found to map in close proximity to significant branching associations.

**Conclusions:**

In sunflower, variation in branching is genetically complex and overall branching patterns (i.e., apical vs. basal) were found to be influenced by distinct genomic regions. Moreover, numerous candidate branching genes mapped in close proximity to significant branching associations. Although the sunflower genome exhibits localized islands of elevated linkage disequilibrium (LD), these non-random associations are known to decay rapidly elsewhere. The subset of candidate genes that co-localized with significant associations in regions of low LD represents the most promising target for future functional analyses.

**Electronic supplementary material:**

The online version of this article (doi:10.1186/s12870-015-0458-9) contains supplementary material, which is available to authorized users.

## Background

Shoot branching is a major determinant of plant architecture and plays an important role in the adaptation of plants to their environment. Variation in branching helps plants compete with their neighbors and also offers protection against herbivory [1-4]. Shoot branching can also affect developmental phenotypes such as flowering time and reproductive success [5]. Moreover, this trait is an important component of the so-called “domestication syndrome” [6], with many crops exhibiting reduced branching (i.e., increased apical dominance) relative to their wild progenitors.

In cultivated sunflower (Helianthus annuus L.), selection during domestication resulted in the production of an apically dominant, unbranched growth form that differs markedly from its highly branched wild progenitor (common sunflower; also *H. annuus*) [7-10]. During the transition to hybrid breeding in the mid-20^th^ century, however, recessive branching was reintroduced to the sunflower gene pool to produce male-fertile restorer (R) lines that can be crossed with unbranched, cytoplasmic male-sterile (i.e., female; A) lines to produce unbranched, fully fertile hybrids. The apical branching of the R-lines is desirable because it provides fertile pollen for a longer time period, resulting in a longer window for pollination and hybrid production [11]. The modern-day cultivated sunflower gene pool thus exhibits substantial variation in plant architecture, making it an ideal system to study the genetics of branching.

In general terms, branching is initiated from axillary meristems in leaf axils on the primary shoot. These meristems give rise to axillary buds which remain dormant or grow out into a branch that can be influenced by environmental conditions or developmental signals such as hormones [5]. Three phytohormones (auxin, cytokinin [CK], and strigolactone [SL]) and genes associated with their homeostasis and signaling are thought to be largely responsible for the regulation of branching [12-15]. Bud outgrowth is inhibited by basipetal transport of auxin produced at the shoot apical meristem. SL is a carotenoid-derived phytohormone that also inhibits bud outgrowth. It is produced in the roots and transported acropetally in the stem [12,16]. In contrast to auxin and SL, CKs are locally synthesized in the bud and promote the outgrowth of the axillary bud. Ultimately, cross talk by these phytohormone related pathways regulates branching [12]. Additionally, genes related to gibberellic acid (GA) and polyamine metabolism, and genes encoding transcription factors, at least one MAP kinase, and cytochrome P450 all play important roles in axillary bud initiation and branch growth [15-18].

In crosses between cultivated and wild sunflower, branching has been found to be a genetically complex trait influenced by numerous small effect loci distributed throughout the genome [19,20]. Classical genetic analyses in cultivated sunflower have, however, revealed the existence of loci with major effects on both apical and basal branching [11,21-23]. More recently, quantitative trait locus (QTL) mapping has been used to localize the recessive apical branching of restorer lines to a region (known as the *B* locus) on the upper half of linkage group (LG) 10 [24,25]. The unbranched phenotype characteristic of female lines and hybrids is thought to be controlled by the dominant *B* allele, while the branched R-lines are homozygous for the recessive *b* allele. While traditional QTL analyses have proven to be useful in identifying genomic regions that influence plant architecture in sunflower, this general approach suffers some limitations. Most notably, the use of biparental populations only enables the analysis of two alleles per gene, and also limits the number of recombination events, thereby providing relatively limited genetic resolution [26].

Association mapping (also known as LD mapping) has emerged as an alternative to QTL mapping for investigating the genetic basis of quantitative traits [27]. Because it involves the analysis of a diverse collection of more or less unrelated individuals, association mapping allows for the simultaneous evaluation of the effects of multiple haplotypes across diverse genetic backgrounds. Moreover, because association populations typically capture numerous generations of historical recombination, this approach provides much higher resolution than is possible with a family-based mapping population. Herein, we report the results of a detailed analysis of variation in branching in an association mapping population that captures nearly 90% of the allelic diversity present within the cultivated sunflower gene pool [28,29]. We evaluated this population for various branching-related traits at three different locations and tested for genetic associations across the genome using genotypic data derived from a high-density SNP array [30,31]. We also identified candidate genes involved in hormonal or transcriptional regulation that mapped in close proximity to significant associations.

## Methods

### Development of the association mapping population

The development and initial characterization of the sunflower association mapping population utilized herein are described by Mandel et al. [28,29]. Briefly, a diverse collection of cultivated sunflower lines was obtained from the USDA North Central Regional Plant Introduction Station (NCRPIS; Ames, IA, USA) and from the French National Institute for Agricultural Research (INRA; France). These lines were genotyped with simple-sequence repeat (SSR) markers distributed across all 17 LGs and the resulting data were used to identify hierarchical subsets of lines that captured maximum diversity [28]. The present study was based on the same subset of 288 lines employed by Mandel et al. [29], which differed slightly from the core 288 described by Mandel et al. [28] due to limited seed availability for some lines. This population, which is available for distribution from the Germplasm Resources Information Network (GRIN) of the National Plant Germplasm System, is known as UGA-SAM1. Of the full set of 288 lines in this population, only 271 were included in our final analyses due to germination difficulties and plant loss during the growing season. These lines capture nearly 90% of the allelic diversity and include lines that are oil and confectionery types from the two major heterotic groups in cultivated sunflower as well as select open-pollinated varieties (OPVs) and land races. Many of the lines were advanced via single-seed descent for one or two cycles to reduce residual heterozygosity prior to the start of this experiment.

### Field design and phenotypic analysis of branching traits

All 288 lines were planted in two replicates using an alpha lattice at three different locations during the spring of 2010. The three locations were: the UGA Plant Sciences Farm, (Oconee County, GA, USA), the NCRPIS at Iowa State University (Ames, IA, USA), and the University of British Columbia’s Botany Gardens (Vancouver, BC, Canada) [29]. Individuals of each line (3–4 individuals per replicate per location) were evaluated at the R9 reproductive stage, which represents physiological maturity [32].

**Figure 1:**
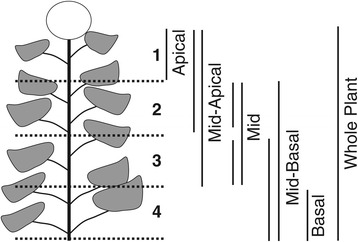
**Branching traits in the association mapping population.** Individual plants were divided into four quarters (1^st^, 2^nd^, 3^rd^, and 4^th^) and the numbers of branches were counted in each quarter. Lines were grouped into specific branching types based on the quarter in which they exhibited branching. Lines were categorized into apical (1^st^ quarter), mid-apical (1^st^ and 2^nd^ quarter; 1^st^, 2^nd^, and 3^rd^ quarter), mid (2^nd^ quarter; 3^rd^ quarter; 2^nd^ and 3^rd^ quarter), mid-basal (2^nd^ and 3^rd^ quarter; 2^nd^, 3^rd^, and 4^th^ quarter), basal (4^th^ quarter) and whole plant branching (1^st^, 2^nd^, 3^rd^, and 4^th^ quarter).

At maturity, each plant was divided into four quarters (1^st^, 2^nd^, 3^rd^, and 4^th^, from top to bottom) and the number of primary branches was counted in each quarter. Primary branches were recorded as present if they were longer than 2 cm and had developed a terminal inflorescence. The numbers of primary branches in each quarter were then used to estimate the extent and type of branching on each plant. Apical branching was estimated as the number of primary branches in the 1^st^ quarter, mid-apical branching was estimated as total number of branches in the 1^st^ and 2^nd^ quarters, and so forth (Figure 1). If a particular quarter did not branch, the leaf axils were examined to determine if axillary bud initiation had occurred. If more than 50% of the nodes in a particular quarter displayed initiation, it was recorded as branch initiation. In addition, the presence or absence of secondary branches was recorded.

For each line, branch numbers and lengths were averaged within replicates prior to analysis. For secondary branching, scores of 0 for absence or 1 for presence were assigned and these values were subsequently averaged, as above. Using PROC GLM in SAS (ver. 9.3; SAS Institute, Cary NC), we found a significant genotype x environment (G x E) interaction (*P* < 0.001). Therefore, all subsequent analyses were performed separately by location, which also made possible the identification of loci that appear to be susceptible to environmental effects (i.e., those that were significant in at least one, but not all locations). In each location, genotypes were treated as fixed effects and block and replicates as random effects. For association mapping of the various branching traits, we used least-squares means (LS means) since block and rep effects were found to be statistically significant (*P* < 0.05).

Correlations between various branching traits and among locations were determined using Spearman’s rank correlation coefficients (*ρ*) in JMP (ver. 9; SAS Institute), and corrected for multiple tests using the sequential Bonferroni correction [33]. Principal component analysis (PCA) was performed to visualize the various branching types (e.g., apical vs. basal branching) within the SAM association mapping population using the *pca* function implemented in the FactoMineR package ver. 1.16 [34] available in the R statistical computing language (ver. 3.1.0) [35].

### Genotyping

As described by Mandel et al. [29], total DNA was extracted from pooled leaf tissue from four individuals of each line using a CTAB extraction protocol [36]. These samples were then genotyped using an Infinium SNP array (Illumina, San Diego, CA) at the Emory University Biomarker Service Center. This array was designed to target polymorphic SNPs from across the sunflower genome. Details about the development of this array have been previously provided by Bachlava et al. [30]. Genome studio ver. 2011.1 (Illumina) was used to make SNP calls and map positions were assigned based on the consensus genetic map of Bowers et al. [31]. Of a total of 9,480 SNPs on the array, 5,788 genetically mapped polymorphic SNPs could be reliably scored as apparently single copy loci in our population [29]. Further, only SNPs with a minor allele frequency of ≥10% (5,359) was used for association mapping analyses [30]. The previously identified *B* locus was not directly used as a marker in this study since its presence or absence was not known for all the lines used in this study. However, markers spanning this region (as determined based on marker position on the sunflower consensus map; Bowers et al. [31]) were included in our analyses.

### Association mapping

Association analyses were performed using TASSEL ver. 3.0 [37]. Because such analyses are prone to false positives due to unrecognized kinship and/or population structure [38], three different mixed models were employed. The first model corrected for kinship (K, estimated using SPAGeDi) [39] only. The second and third models corrected for both kinship and population structure. In the Q + K model, population structure (Q) was estimated using STRUCTURE ver. 2.2 [40]. In the P + K model, population structure (P) was estimated via a principal coordinate analysis (PCoA) using GenAlEx ver. 6.41 [41]. Details of the underlying SPAGeDi, STRUCTURE, and PCoA analyses can be found in Mandel et al. [29].

Following the association analyses, Q-Q (quantile-quantile) plots were constructed for each of the three models and compared to the results of a naïve model to select the most appropriate model for analysis. This was performed for apical (1^st^ quarter), mid (2^nd^ and 3^rd^ quarter) and basal (4^th^ quarter) branching. For association mapping of apical branching, the total number of branches in the first quarter from lines that displayed apical, mid-apical, and whole plant branching were included in the analysis. Similarly for all other branching types, the number of branches from all lines that displayed branching in the respective quarter were included in our analyses. Because non-independence of linked markers can result in highly conservative significance thresholds [42], we set a threshold -log(*P*) value of 3.6 (alpha=0.05, *P*=0.00025, log 1/*P*=3.60) to identify significant associations using a multiple testing correction method that accounts for correlation among markers while also controlling the type I error rate [43].

### Identification and mapping of candidate branching genes

In order to identify putative sunflower orthologs of branching genes identified in other plant species (i.e., *Arabidopsis*, rice, pea, and petunia), we searched the literature for genes involved in axillary meristem initiation and outgrowth. These genes included transcription factors such as *REVOLUTA* (*REV*), *LATERAL SUPPRESSOR* (*LAS*) and *REGULATORS OF AXILLARY MERSITEMS* (*RAX 1*, *2*, and *3*) from *Arabidopsis* that have been shown to play important roles during initiation of axillary meristem and bud formation [44-46]. In addition, genes associated with homeostasis and signaling of phytohormones and growth regulators such as auxin, CK, SL, GA, and polyamines were included [12-15,17,18]. Many other genes involved in branch outgrowth that encode transcription factors, cytochrome P450, *MAP KINASE KINASE 7* (*MAPKK7*), arabinogalactan proteins, and other DNA binding proteins were also included. For genes that were a part of a multi-gene family, only the genes that have been implicated in branching have been included. This resulted in the identification of 48 candidate genes (Additional file 1).

Once identified, the sequence of each candidate gene was searched against v0.1 of the draft sunflower genome assembly (http://www.sunflowergenome.org/) using tblastx with an E-value threshold of 1e-6. Since sunflower has undergone at least three whole genome duplication events [47] and is also likely to have experienced numerous segmental duplications, up to eight sunflower blast hits were considered for each of the candidate genes to allow for multiple possible homologs. Map positions of as many of these genes as possible were then determined by comparison to genetically mapped contigs from the whole genome assembly using the whole genome shotgun (WGS) sequence-based sunflower genetic map [48]. Genetic positions from the WGS map were translated into map positions on the Bowers et al. [31] consensus genetic map using common markers. This allowed us to determine if any of the candidate genes mapped in close proximity to SNPs associated with the branching traits. We used a window size of 2.5 cM to determine co-localization since the SNPs in question had been previously ordered using multiple mapping populations differing from the population used to order the WGS map. As a result, an uncertainty of several cM in the exact genetic position of markers remained after map integration. The sunflower contigs containing the candidate genes (based on the blast results) and their position on LGs are listed in Additional file 2. To determine if candidate genes and significant associations occurred in regions of high or low LD, *r*^*2*^ values were computed using the diversity panel between all SNP pairs within 2.5 cMs of each other. An overall *r*^*2*^ value was then computed for each SNP by averaging the individual pairwise *r*^*2*^ values.

## Results

### Association mapping of branching patterns

**Figure 2:**
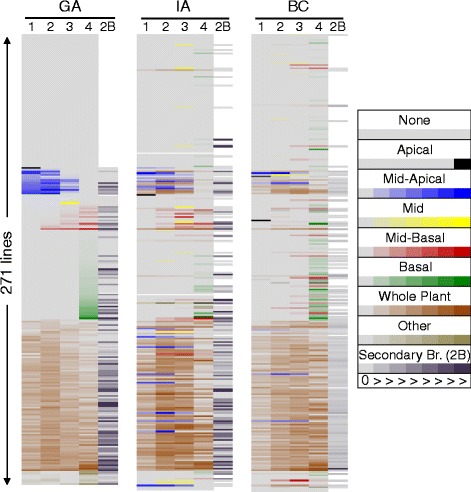
**Branching patterns across the three locations.** A heat map was generated to visualize the type of branching in the same lines across all the three locations. Shown in columns are the number of branches in the 1^st^ quarter (1), 2^nd^ quarter (2), 3^rd^ quarter (3), 4^th^ quarter (4), and secondary branches (2B). Each row displays data for an individual line. Lines have been color coded according to their branching pattern. Color codes are described on the figure; the gradation of color from left to right indicates no branches (or fewer branches in case of whole plant branching) to maximum number of branches observed for a particular type within that location. Lines have been grouped together according to the branching pattern exhibited in Georgia (GA); corresponding lines in Iowa (IA) and British Columbia (BC) are matched up against their position in the GA columns, emphasizing variation across location.

Phenotypic analyses revealed extensive variation in the type and extent of branching within the association mapping population (Table 1, Figure 2, Additional file 3). Depending on the location, 89–102 lines exhibited whole-plant branching, while 70–110 lines exhibited no branching. The numbers of lines that exhibited other types of branching are listed in Table 1. There was high overlap of 77 lines exhibiting whole plant branching at all three locations (Table 2). However, all other branching patterns exhibited extensive variation across locations also displayed as a heat map (Figure 2). There was a greater overlap of lines exhibiting basal branching in GA and BC compared to IA, where many did not branch. Collectively these data illustrate that the environment can exert a strong influence on branching patterns. Interestingly, unbranched lines did not display any axillary bud initiation.

**Table 1:** **Branching patterns across locations (GA, IA, and BC)**

**BRANCHING**	**GA**	**IA**	**BC**
None	79	110	70
Basal	53	24	60
Mid-basal	15	15	18
Mid	6	12	9
Mid-apical	15	16	6
Apical	1	1	1
Whole plant	90	89	102
Other types	8	1	5
Missing data	4	3	0

Total number of lines exhibiting a specific branching type was calculated for each location. “Other types” includes lines that did not fall into standard branching categories such as branching in 1^st^ and 4^th^ quarter or 2^nd^ and 4^th^ quarter or 1^st^, 2^nd^ and 3^rd^ quarter or 1^st^, 2^nd^ and 4^th^ quarter.

**Table 2:** **Similarity in branching patterns across locations**

**Location**	***Branching traits***							
	**None**	**Apical**	**Mid-apical**	**Mid**	**Mid-basal**	**Basal**	**Whole plant**	**Other**
GA, IA, BC	46	-	2	-	1	9	77	-
GA, BC	5	-	1	1	2	24	8	-
GA, IA	19	-	4	-	5	4	-	-
IA, BC	13	-	2	-	2	6	8	-
GA	9	1	8	5	7	16	5	8
BC	6	1	1	8	13	21	9	5
IA	32	1	8	12	7	5	4	1

Summary of the numbers of lines exhibiting similar branching patterns across the three locations (GA, Georgia; IA, Iowa; BC, British Columbia). Several lines had similar branching patterns only across two locations or had a different pattern of branching at every location.

**Figure 3:**
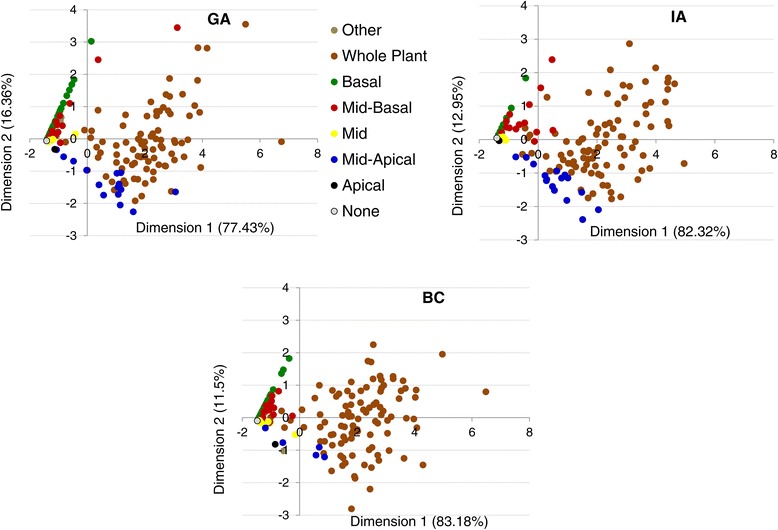
**Principal component analyses (PCA) showing clusters of various branching types in the association mapping population.** The first dimension accounted for 77.4%, 82.3%, and 83.1% of the observed variation in GA, IA, and BC, respectively. The second dimension accounted for 16.4%, 13.0%, and 12.0% of the variation observed in GA, IA, and BC, respectively. Each dot represents a line and the color of the dot represents the type of branching.

As expected, the correlation analyses revealed positive correlations amongst branching types and across locations (Table 3). However, basal branching tended to exhibit lower correlations overall, both within and across locations. This suggests that basal branching may be under different genetic control, and might also be more subject to environmental influences than apical and mid branching. In the PCA (Figure 3), the first dimension accounted for 77-83% (depending on location) of the underlying variation, and largely reflected variation in the extent of branching (i.e., the number of branches produced). The second dimension captured 12-16% of the phenotypic variation and primarily reflected differences in apical vs. basal branching.

**Table 3:** **Correlation of apical, mid, and basal branching across GA, IA, and BC**

**Location**		**GA**				**IA**				**BC**			
**Branching**		**Apical (1Q)**	**Mid (2Q)**	**Mid (3Q)**	**Basal (4Q)**	**Apical (1Q)**	**Mid (2Q)**	**Mid (3Q)**	**Basal (4Q)**	**Apical (1Q)**	**Mid (2Q)**	**Mid (3Q)**	**Basal (4Q)**
**GA**	Apical (1Q)	-	0.91	0.81	0.51	0.90	0.89	0.82	0.60	0.90	0.88	0.85	0.63
	Mid (2Q)		-	0.88	0.57	0.88	0.89	0.85	0.64	0.88	0.89	0.87	0.65
	Mid (3Q)			-	0.65	0.81	0.86	0.85	0.67	0.84	0.84	0.82	0.65
	Basal (4Q)				-	0.52	0.59	0.60	0.69	0.56	0.56	0.58	0.74
**IA**	Apical (1Q)					-	0.93	0.86	0.64	0.90	0.89	0.86	0.64
	Mid (2Q)						-	0.93	0.72	0.88	0.89	0.88	0.70
	Mid (3Q)							-	0.78	0.83	0.86	0.87	0.71
	Basal (4Q)								-	0.62	0.64	0.68	0.76
**BC**	Apical (1Q)									-	0.92	0.88	0.66
	Mid (2Q)										-	0.92	0.66
	Mid (3Q)											-	0.74
	Basal (4Q)												-

The correlation between each branching type was calculated within and across locations (GA, Georgia; IA, Iowa; BC, British Columbia). All values were positive and significant (*P* < 0.0001) after correcting for multiple comparisons. Q: plant quarter.

### Association analyses

**Figure 4:**
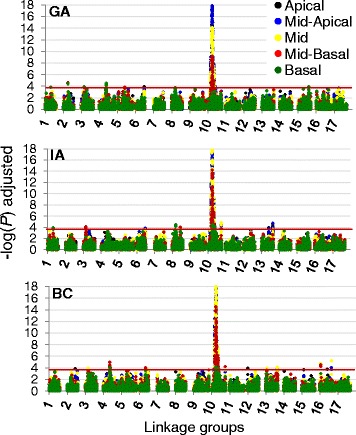
**Manhattan plots of association results across linkage groups for each location.** The negative log of the *P*-values (adjusted) for association tests using each SNP from across the genome was plotted against genomic position. Each dot represents a SNP and the color of the dot represents the type of branching being analyzed. The red line indicates the significance threshold (3.6), above which the associations with SNPs are considered significant. This value reflects a multiple-testing correction following the method by Gao et al. [43].

The P + K model, which corrected for both kinship and population structure (using PCoA results) appeared to be the most conservative model across traits and showed the lowest tendency toward false positives (Additional file 4). This model was thus selected for all subsequent association analyses. Our analyses detected significant associations on multiple LGs (Table 4). In total, SNPs on 14 of the 17 sunflower LGs groups (all but LGs 7, 11, and 14) showed significant associations with various branching types. The Manhattan plots (Figure 4) illustrate our results for the various branching types at all three locations. Overall, the largest number of significant SNPs could be found on LG 10 in a region that was mostly associated with branching in the apical and mid regions of the plant. The broad peak of associations that is visible on the upper portion of LG 10 (referred to herein as 10a; Table 4; Figure 4) corresponds to the so-called *B* locus. In general, we were able to identify SNPs associated with basal and mid-basal branching which were distinct from apical, mid-apical, and mid branching (Table 4; Figure 4). For example, certain LGs (such as LG 4 in GA and BC and LG 8 in GA and IA) were associated with basal and mid-basal branching whereas SNPs near the bottom of LG 6 (in GA, IA and BC) and 13 (IA and BC) were associated with branching in the apical and mid regions. Only one secondary branching association was observed on LG 10 (in BC). The results for whole plant branching are not presented here, as that information was analyzed by Mandel et al. [29].

**Table 4:** **Linkage groups associated with distinct branching patterns**

**Quarter**	**1**	**12**	**123**	**2**	**3**	**23**	**34**	**234**	**4**	
**Branching**	**Apical**	**Mid-apical**	**Mid-apical**	**Mid**	**Mid**	**Mid**	**Mid-basal**	**Mid-basal**	**Basal**	**2B** ^**a**^
**1**										
**2a**										
**2b**										
**3**										
**4**										
**5**										
**6a**										
**6b**										
**8a**										
**8b**										
**9**										
**10a**										
**10b**										
**12**										
**13a**										
**13b**										
**13c**										
**15a**										
**15b**										
**16a**										
**16b**										
**16c**										
**17**										

Plants were classified into various branching types depending on the presence of branches in a given quarter (see Figure 1 for more details). ^a^2B indicates secondary branching. The orange (Georgia), black (Iowa), and blue (British Columbia) Xs indicate significant SNPs at the three different locations. When more than one association was detected on a single linkage group (LG), the associations were labeled alphabetically based on their position in the LG. The position of the *B* locus corresponds to the associations on LG 10a (see methods for details).

### Identification of candidate genes associated with various branching phenotypes

A total of 48 candidate branching genes were identified from other species (Additional file 1), and homologs to 39 of these genes could be identified in sunflower. This attrition is likely due to the use of an incomplete, draft assembly of the genome. We allowed up to eight of the best blast hits for each gene which resulted in 278 homologs. Of these, 153 (corresponding to 38 genes) could be placed on the sunflower genetic map (Additional files 2 and 5). Among these 153 blast hits, 92 unique contigs were identified. In many cases, multiple genes (belonging to gene families) were found within the same sunflower contig.

**Figure 5:**
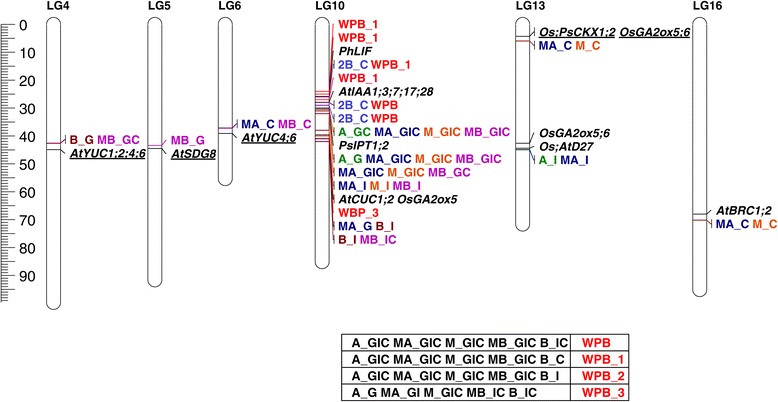
**Co-localization of SNPs associated with branching-related traits and candidate branching genes.** Only SNPs mapped in close proximity to putative branching genes exhibiting significant associations with branching are displayed. The SNP-associated branching traits are apical (A; in green), mid-apical (MA; in dark blue), mid (M; in orange), mid-basal (MB; in magenta), basal (B; in brown), and secondary branching (2B; in light blue). Location information is given following an underscore (G=Georgia, I=Iowa, and C=British Columbia). SNPs associated with all branching traits except 2B are presented as whole plant branching (WPB) and the numbers indicate differences among geographical locations for a given branching type (for LG 10). Gene names are shown in black, *YUCCA* (*YUC*); *AUXIN/INDOLE-3-ACETIC ACID* (*IAA*); *ISOPENTYLTRANSFERASE* (*IPT*); *CK OXIDASE/DEHYROGENASE* (*CKX*); *GIBBERELLIN 2-OXIDASES* (*GA2ox*); *DWARF27* (*D27*); *CUP-SHAPED COTYLEDON* (*CUC*); *LATERAL SHOOT INDUCING FACTOR* (*LIF*); *BRANCHED2* (*BRC2*); *SET DOMAINGROUP8* (*SDG8*). Underlined genes lie in regions of low LD. *At*, *Ph*, *Ps*, and *Os* stand for *Arabidopsis thaliana*, *Petunia x hybrida*, *Pisum sativum*, and *Oryza sativa*, respectively.

**Figure 6:**
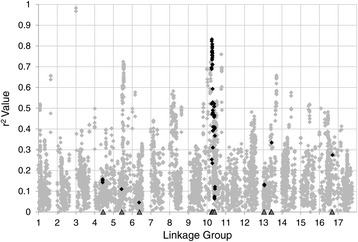
**Genome-wide summary of LD.** To investigate the extent of LD across the genome, *r* ^*2*^ values were computed between all possible pairs of SNPs within a 2.5 cM window. An average *r* ^*2*^ value was then calculated for each SNP (grey) and ordered along the x-axis as a function of genomic position. SNPs that were significantly associated with branching and located within 2.5 cM of candidate genes are colored in black. The positions of the candidate genes are indicated by a grey arrow on the x-axis. See Additional file 6 for a more detailed view, including gene names.

In comparing the map positions of these genes to the locations of SNPs exhibiting significant associations, we identified 13 potential candidate branching genes (from the larger set of 92 unique genes) that mapped in close proximity to various branching traits (Figure 5). Genes of interest involved in hormone related pathways included an auxin biosynthetic gene (*YUCCA*; *YUC*), an auxin induced gene (*AUXIN/INDOLE-3-ACETIC ACID*; *Aux/IAA*), and genes involved in CK biosynthesis (*ISOPENTYLTRANSFERASE*; *IPT*) and degradation (*CK OXIDASE/DEHYDROGENASE*; *CKX*), GA catabolism (*GIBBERELLIN 2-OXIDASES*; *GA2ox*), and SL biosynthesis (*DWARF27*; *D27*)*.* Several transcription factors also co-localized with significant branching associations, including *CUP-SHAPED COTYLEDON* (*CUC*), *LATERAL SHOOT INDUCING FACTOR* (*LIF*), *BRANCHED* (*BRC*), as well as a histone methyltransferase, *SET DOMAIN GROUP8* (*SDG8*). Of these, eight candidate genes were in regions of elevated LD (Figures 5 and 6; Additional file 6), including all genes on LG 10, one on the lower half of LG 13, and one on LG 16. The remaining genes on LGs 4, 5, 6, and the upper half of 13 were present in regions of lower LD (i.e., *r*^2^ < 0.20).

## Discussion

The identification of loci influencing specific branching patterns has the potential to facilitate the manipulation of plant architecture, which can influence yield and seed/fruit quality in crops [15,49-52]. Here, we have identified numerous distinct loci that influence apical vs. basal branching in sunflower. It is important to note, however, that the occurrence of variation in branching patterns within lines across the three locations suggests that environmental variation also plays an important role in determining sunflower branching architecture. This conclusion is consistent with the results of previous studies on the effect of external factors on branch formation. For example, increased planting density can result in a suppression of branching [53,54], and photoperiod is also known to affect branching [55-58]. Other environmental factors influencing branching include light levels and quality [59-61], plant nutrition status, and availability of nutrients [62,63]. It seems likely that some combination of these factors influenced our results across locations.

The formation of a branch involves two developmental processes: the initiation of an axillary bud and its elongation into a branch [5,64]. As noted above, genes such as *REV*, *LAS*, and *RAX* in *Arabidopsis* and their orthologs in rice and tomato play a role during axillary meristem initiation and bud formation [44-46,65-67]. Because the non-branching lines in our study also did not exhibit axillary bud initiation, we were unable to separate initiation from outgrowth and were thus unable to identify loci contributing specifically to one process or the other. The association mapping population did, however, exhibit substantial diversity in branching patterns which facilitated the identification of multiple branching-related loci that had not been previously identified in sunflower. These included associations on LGs 1, 2b, 5, 12, 14, 15, and 16 not previously identified via traditional QTL mapping [19,20,68] or via association mapping [29].

Importantly, our results also revealed that apical and basal branching are under largely independent genetic control (Figure 4; Table 4). Previous studies have identified a small number of loci that influence either apical or basal branching [22,23] and suggested that these traits are controlled by different loci, but our results indicate that the effects of branching-related loci are largely compartmentalized in sunflower. In fact, with the exception of loci on LGs 6a, 10a, and 10b, all three of which are associated with whole plant branching, the remaining 20 significant associations had primarily apical or basal effects. As mentioned above, the broad peak of associations on LG 10a (Figure 4) corresponds to the so-called *B* locus [11]. This locus is responsible for the branching that was reintroduced into the sunflower gene pool to extend the flowering time in R lines, and is present as a large haplotypic block within many of these lines [29], resulting in a sizable island of elevated LD (Figure 6). Similar instances of elevated LD, mostly associated with past episodes of selection during the evolution of cultivated sunflower, are also visible elsewhere in the genome [29]. While multiple novel branching loci were identified in this study, it is possible that segregation of the *B* locus masked the effect of other loci contributing to the variation observed within this population. The analysis of a population that varies for branching while being fixed for the recessive, unbranched *b*/*b* genotype at this locus has the potential to shed light on this possibility.

We further identified candidate branching genes that are co-localized with significant branching associations. Similar approaches using association mapping have been used to identify positional candidates for important agronomic traits in crops such as rice (e.g., [69,70]) and maize (e.g., [71]), to identify candidate genes associated with flowering time, pathogen resistance, and tolerance to salinity in *Arabidopsis* (e.g., [72,73]). Of course, the resolution afforded by association mapping varies across the genome due to localized variation in the extent of LD. We thus focused on the identification of candidate genes that co-localized with branching associations in regions of low LD (i.e., *r*^2^ < 0.20). These genes included homologs of *AtYUC* that were closely linked to mid-basal and basal branching on LG 4 and mid-apical and mid-basal branching on LG 6; a homolog of *CKX*, which co-localized with apical and mid-branching on LG 13; and homologs of *GA2ox5* and *GA2ox6* in that same region of LG 13. In addition to these hormone-related genes, a homolog of *SDG8* that was associated with mid-basal branching co-localized on LG 5. *SDG8* is thought to epigenetically regulate other branching genes, and loss of this gene in *Arabidopsis* exhibit increased shoot branching [74]. The above genes are thus excellent candidates for further functional characterization. In maize, the primary determinant of branched vs. unbranched is *teosinte branched1* (*tb1*), which is a transcription factor within the *CYCLOIDEA* (*CYC*)/*TB1* subfamily of the TEOSINTE-BRANCHED1/CYCLOIDEA/PCF (TCP) transcription factor family [75-77]. While homologs of this gene are known to influence branching in other species such as rice [78] and *Arabidopsis* [54], we found no evidence for a similar role of *CYC*-like genes in sunflower (see also Chapman et al. [79]).

## Conclusions

In this study, we identified numerous loci with significant effects on branching in sunflower, many of which had variable effects across environments. This includes multiple genomic regions that had not previously been implicated in branching. Interestingly, the majority of these loci primarily affected either apical or basal branching, as opposed to influencing branching at the whole-plant level. We also identified a collection of branching-related candidate genes that co-localized with significant association in regions of low LD, providing us with a pool of promising candidates for future functional validation.

## Additional files

Additional file 1:
**Candidate branching genes.** Genes involved in axillary meristem initiation and branch outgrowth in *Arabidopsis thaliana* (*At*), *Petunia* x *hybrida* (*Ph*), *Pisum sativum* (*Ps*), and *Oryza sativa* (*Os*). Additional file 2:
**List of candidate genes with significant hits to contigs within the draft sunflower genome assembly.** For each candidate gene, up to eight sunflower blast hits were considered, with the actual number being indicated as the hit number. The positions of the mapped genes within the contigs are also listed. See text for details. Additional file 1 about the candidate genes. Additional file 3:
**Phenotypic data for branching in the association mapping population.** Also included are the classification (i.e., oil vs. nonoil, breeding group, etc.) and USDA designation of each line. For more specific descriptions of these lines, refer to Mandel et al. [28]. Locations are G=Georgia, I=Iowa, and C=British Columbia. Q indicates quarter. The various branching types are illustrated in Figure 1. Additional file 4:
**Quantile-quantile (Q-Q) plots of branching associations based on models accounting for the effects of kinship and/or population structure vs. a naive model.** Results for branching in each of the four quarters at all three locations were computed using three models, K (red), P + K (blue), and Q + K (grey) and compared against the results of a naive model (brown). The expected values are shown in green. Locations are indicated as G=Georgia, I=Iowa, and C=British Columbia. Q indicates quarter. The branching types are illustrated in Figure 1. Additional file 5:
**Sunflower genetic map depicting the positions of significant SNPs associated with branching traits and candidate branching genes.** Shown are all 17 LGs and positions of all significant SNPs found in the study. Candidate branching genes are in black. Branching traits associated with the SNPs are color coded as in Figure 5 with apical (A; in green), mid-apical (MA; in dark blue), mid (M; in orange), mid-basal (MB; in magenta), basal (B; in brown), and secondary branching (2B; in light blue). Locations are indicated as follows: G=Georgia, I=Iowa, and C=British Columbia). For LG 10 only, SNPs associated with all branching traits other than 2B are indicated as whole plant branching (WPB) and the numbers denote differences among geographical locations for a given branching type. Additional file 6:
**Average** ***r*** ^***2***^ **values for SNPs along LGs that contained candidate branching genes that co-localized with significant branching associations.** Average *r* ^*2*^ values were calculated as described in Figure 6. SNPs exhibiting significant branching associations and located in close proximity to a candidate branching gene are depicted in red. The positions of the putative candidate genes are indicated by blue arrows on the x-axis; letters refer to specific genes.

## Abbreviations

A lines: Cytoplasmic male-sterile female lines
*Aux/IAA*: *AUXIN/INDOLE-3-ACETIC ACID*
*BRC*: *BRANCHED*
CK: Cytokinin
*CKX*: *CYTOKININ OXIDASE/DEHYDROGENASE*
*CUC*: *CUP-SHAPED COTYLEDON*
*CYC*: *CYCLOIDEA*
*D27*: *DWARF27*
GA: Gibberellic acid
*GA2ox*: *GIBBERELLIN 2-OXIDASES*
GRIN: Germplasm Resources Information Network
INRA: French National Institute for Agricultural Research
*IPT*: *ISOPENTYLTRANSFERASE*
K: Kinship
*LAS*: *LATERAL SUPPRESSOR*
LD: Linkage disequilibrium
LG: Linkage group
*LIF*: *LATERAL SHOOT INDUCING FACTOR*
LS Means: Least-squares means
*MAPKK7*: *MAP KINASE KINASE 7*
NCRPIS: North Central Regional Plant Introduction Station
OPVs: Open-pollinated varieties
P: Population structure estimated using principal coordinate analysis
PCA: Principal component analysis
PCoA: Principal coordinate analysis
Q: Population structure estimated using STRUCTURE
Q-Q plots: quantile-quantile plots
QTL: Quantitative trait locus
R lines: Male-fertile restorer lines
*RAX*: *REGULATORS OF AXILLARY MERSITEMS*
*REV*: *REVOLUTA*
*SDG8*: *SET DOMAIN GROUP8*
SL: Strigolactone
SSR: Simple-sequence repeat
*tb1*: *teosinte branched 1*
TCP: TEOSINTE-BRANCHED1/CYCLOIDEA/PCF
WGS: Whole genome shotgun
YUC: YUCCA
