# Supplementary material for: Association mapping in sunflower (Helianthus annuus L.) reveals independent control of apical vs. basal branching
Source: BMC Plant Biol. 2015 Mar 11;15:84. doi: 10.1186/s12870-015-0458-9 (PMC4407831; doi:10.1186/s12870-015-0458-9)
Supplement: Additional file 1: — Candidate branching genes. Genes involved in axillary meristem initiation and branch outgrowth in Arabidopsis thaliana (At), Petunia x hybrida (Ph), Pisum sativum (Ps), and Oryza sativa (Os). [file 12870_2015_458_MOESM1_ESM.pdf]

Additional file 3

GENE	ABBREVIATION	DESCRIPTION	REFERENCE
Transcription factors and other branching related genes			
REVOLUTA	*AtREV*	Homeodomain leucine zipper transcription factor	[45]
LATERAL SUPPRESSOR	*AtLAS*	GRAS family of transcription factor	[44; 65-66]
REGULATOR OF AXILLARY MERISTEMS 1; 2; 3	*AtRAX1; 2; 3*	R2R3 class of MYB transcription factor	[46; 67]
SUPERSHOOT	*AtSPS1*	Cytochrome P450	[80]
BRANCHED 1; 2	*AtBRC1; 2*	TCP transcription factor	[54; 59; 76; 78]
CUP-SHAPED COTYLEDON 1; 2; 3	*AtCUC1; 2; 3*	NAC domain transcription factor	[81-82]
LAX PANICLE	*OsLAX*	Basic helix–loop–helix transcription factor	[83]
BUSHY AND DWARF1	*AtBUD1*	MAP KINASE KINASE 7	[84]
METHYL CPG BINDING 9	*AtMBD9*	CpG-binding domain (MBD)-containing protein	[85-86]
SET DOMAIN GROUP 8	*AtSDG8*	Histone N-Methyltransferase	[87]
ARABINOGALACTAN PROTEIN -1	*LeAGP-1*	Arabinogalactan-proteins	[88]
LATERAL SHOOT INDUCING FACTOR	*PhLIF*	Zinc-finger protein	[89]
Strigolactone-related genes			
MORE AXILLARY GROWTH 1	*AtMAX1*	Cytochrome P450	[58]
MORE AXILLARY GROWTH 2	*AtMAX2*	F-Box Leucine rich repeat protein	[58; 90-91]
MORE AXILLARY GROWTH 3	*AtMAX3*	Carotenoid cleaving dioxygenases 7	[91-94]
MORE AXILLARY GROWTH 4	*AtMAX4*	Carotenoid cleaving dioxygenases 8	[95-98]
DWARF27	*OsD27; AtD27*	Iron-containing protein	[99-100]
Cytokinin-related genes			
ADENOSINE PHOSPHATE-ISOPENTENYLTRANSFERASE 1; 2	*PsIPT1; 2*	Adenylate isopentenyltransferase, biosynthesis	[14]
CYTOKININ OXIDASE/DEHYDROGENASE1; 2	*PsCKX1; 2; OsCKX2*	Cytokinin oxidase/dehydrogenase, degradation	[14; 101]
LONELY GUY 2; 4; 7	*AtLOG2; 4; 7; OsLOG*	Lysine decarboxylase; cytokinin-activating enzyme	[102-103]
Auxin-related genes			
INOSITOL POLYPHOSPHATE KINASE 2 BETA	*AtIPK2B*	Inositol polyphosphate 3-/6-/5-kinase	[104]
AUXIN RESISTANCE 1	*AtAXR1*	Related to ubiquitin-activating enzyme	[105-106]
INDOLE-3-ACETIC ACID INDUCIBLE 1; 3; 7; 17; 28	*AtIAA1; 3; 7; 17; 28*	Auxin responsive, TxF	[107-111]
PIN-FORMED 1	*PsPIN1*	Auxin efflux carrier	[14;16]
PINOID	*AtPID*	Serine/threonine kinase	
YUCCA 1; 2; 4; 6	*AtYUC1; 2; 4; 6*	Flavin monooxygenase	[112-113]
GA-related genes			
GIBBERELLIN 2-OXIDASE 5; 6	*OsGA2ox5; 6*	Gibberellin 2-oxidases, GA catabolism	[17]
Polyamine-related gene			
BUSHY AND DWARF 2	*AtBUD2*	Adenosylmethionine decarboxylase	[18]
