# Supplementary figures and images for: Association mapping in sunflower (Helianthus annuus L.) reveals independent control of apical vs. basal branching
Source: BMC Plant Biol. 2015 Mar 11;15:84. doi: 10.1186/s12870-015-0458-9 (PMC4407831; doi:10.1186/s12870-015-0458-9)

Additional file 2

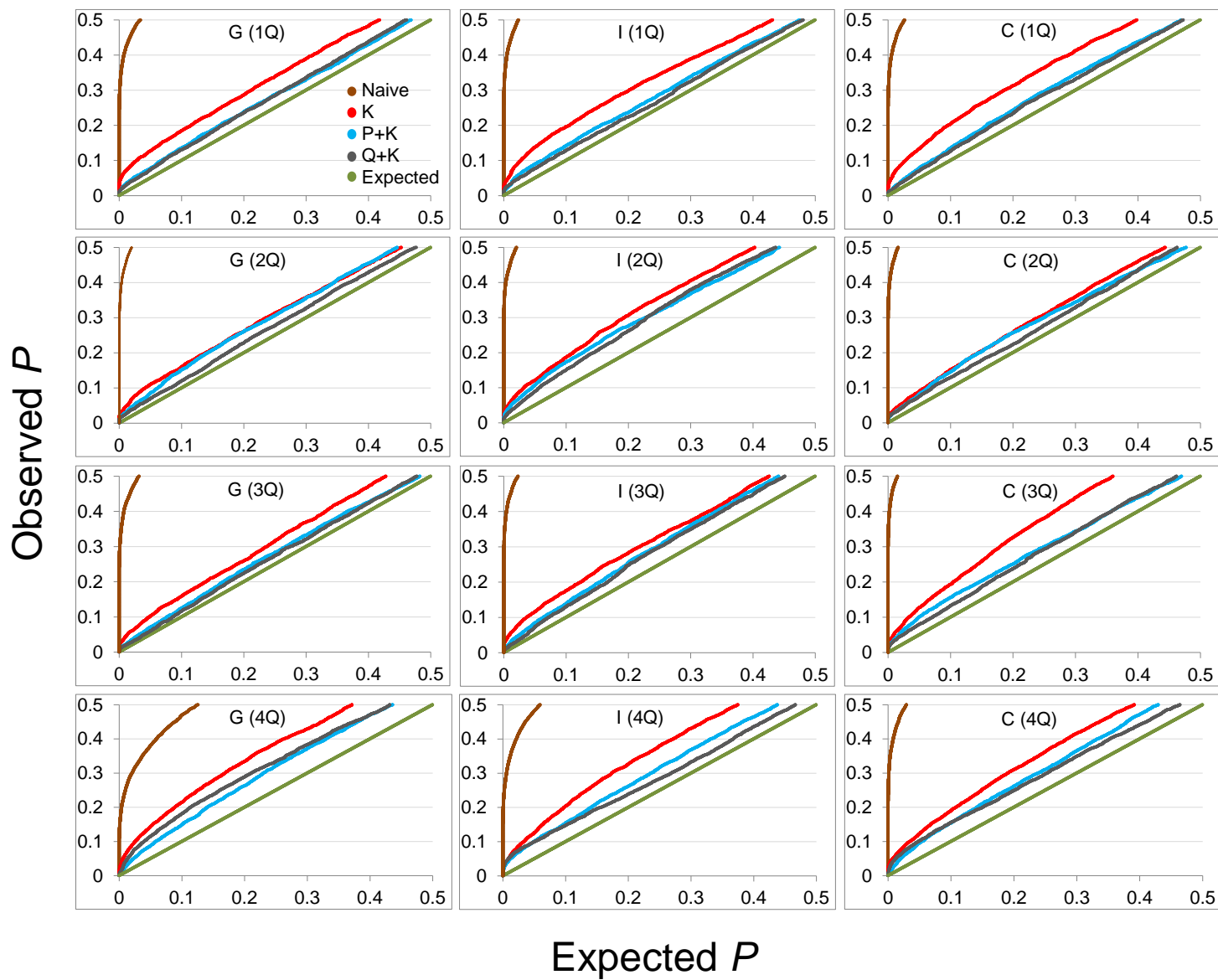

Supplement: Additional file 4: — Quantile-quantile (Q-Q) plots of branching associations based on models accounting for the effects of kinship and/or population structure vs. a naive model. Results for branching in each of the four quarters at all three locations were computed using three models, K (red), P + K (blue), and Q + K (grey) and compared against the results of a naive model (brown). The expected values are shown in green. Locations are indicated as G=Georgia, I=Iowa, and C=British Columbia. Q indicates quarter. The branching types are illustrated in Figure 1. [file 12870_2015_458_MOESM4_ESM.pdf]

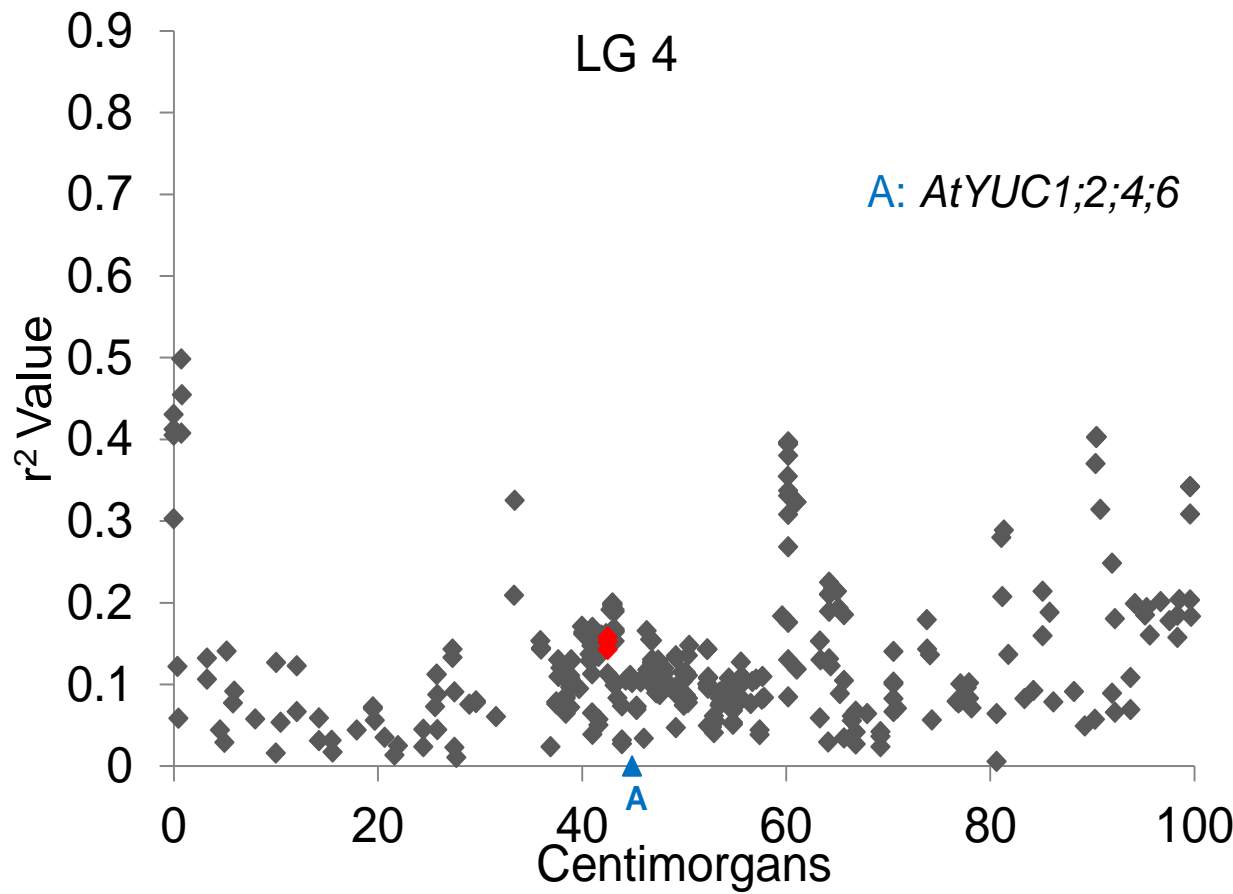

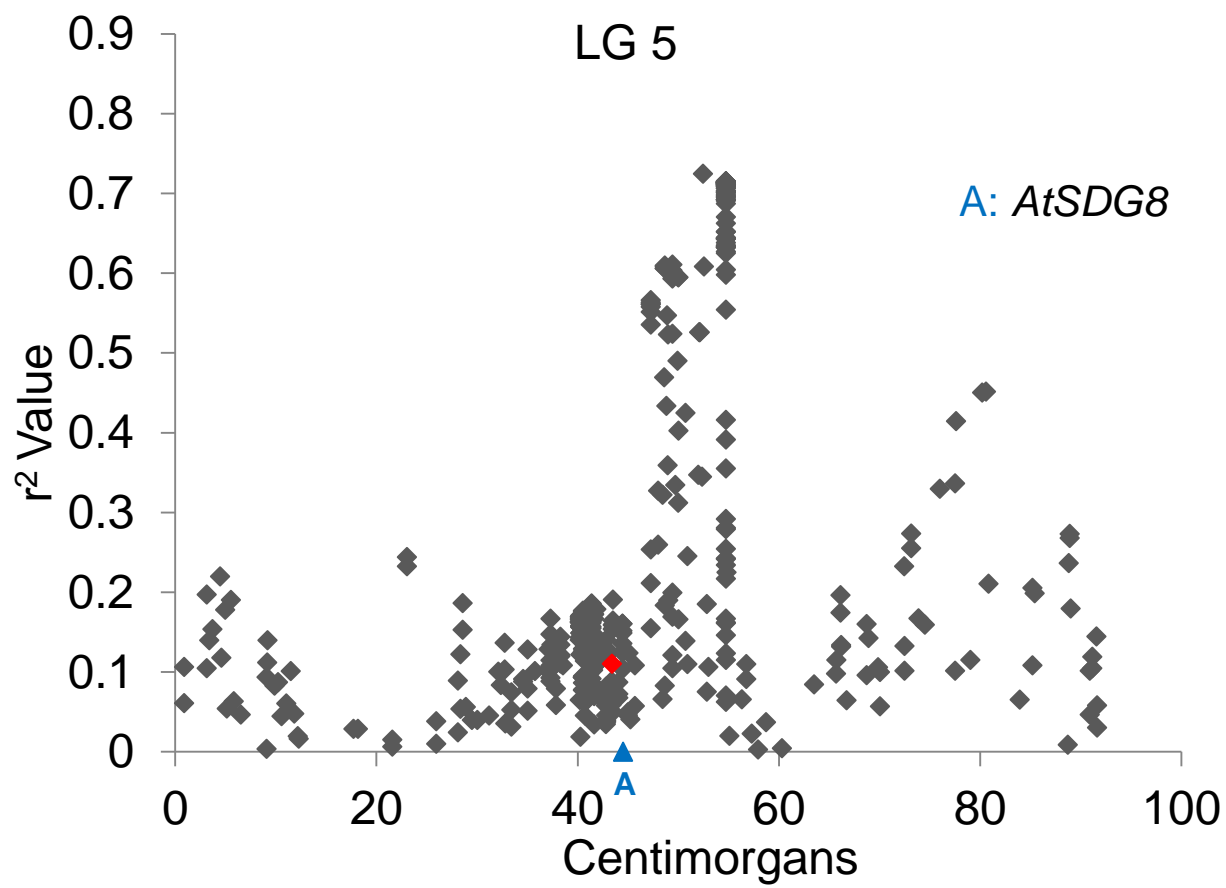

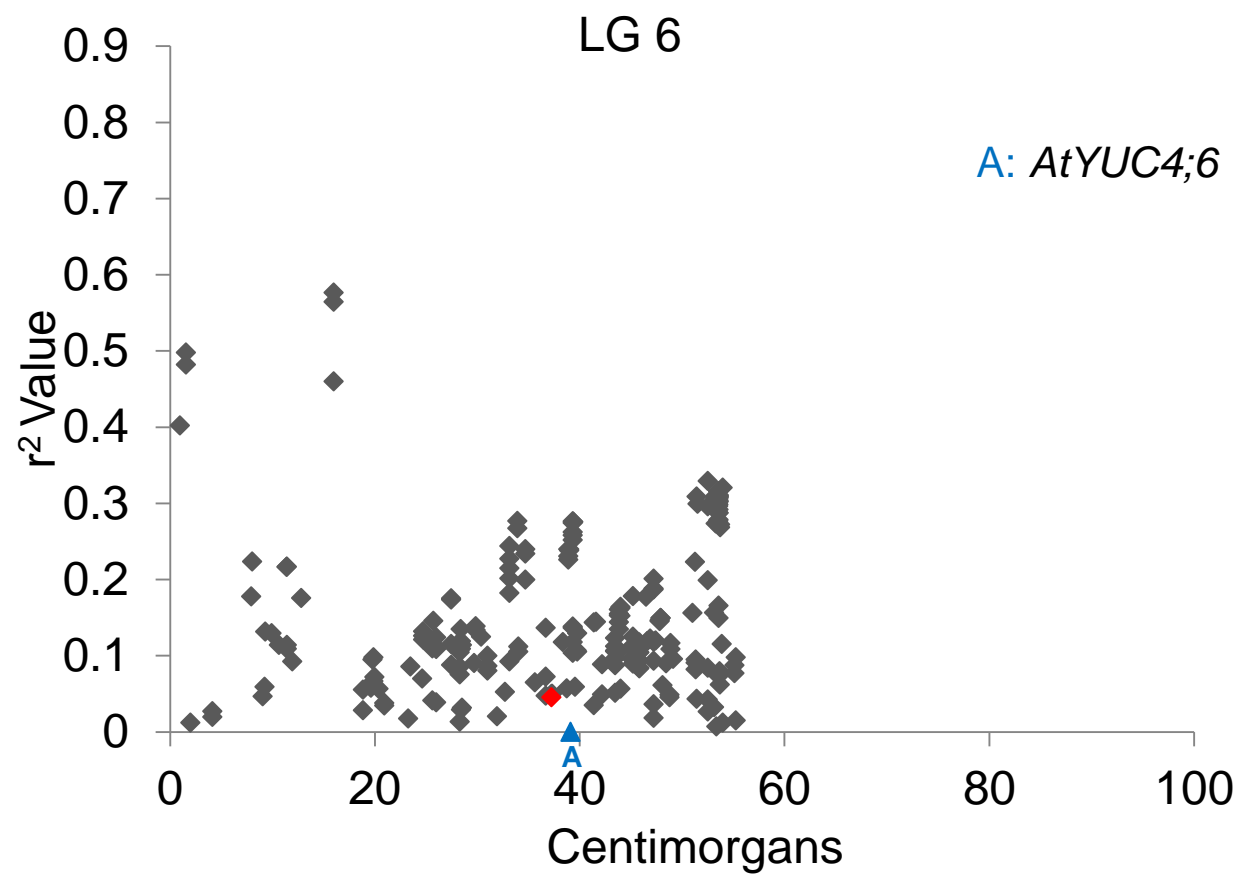

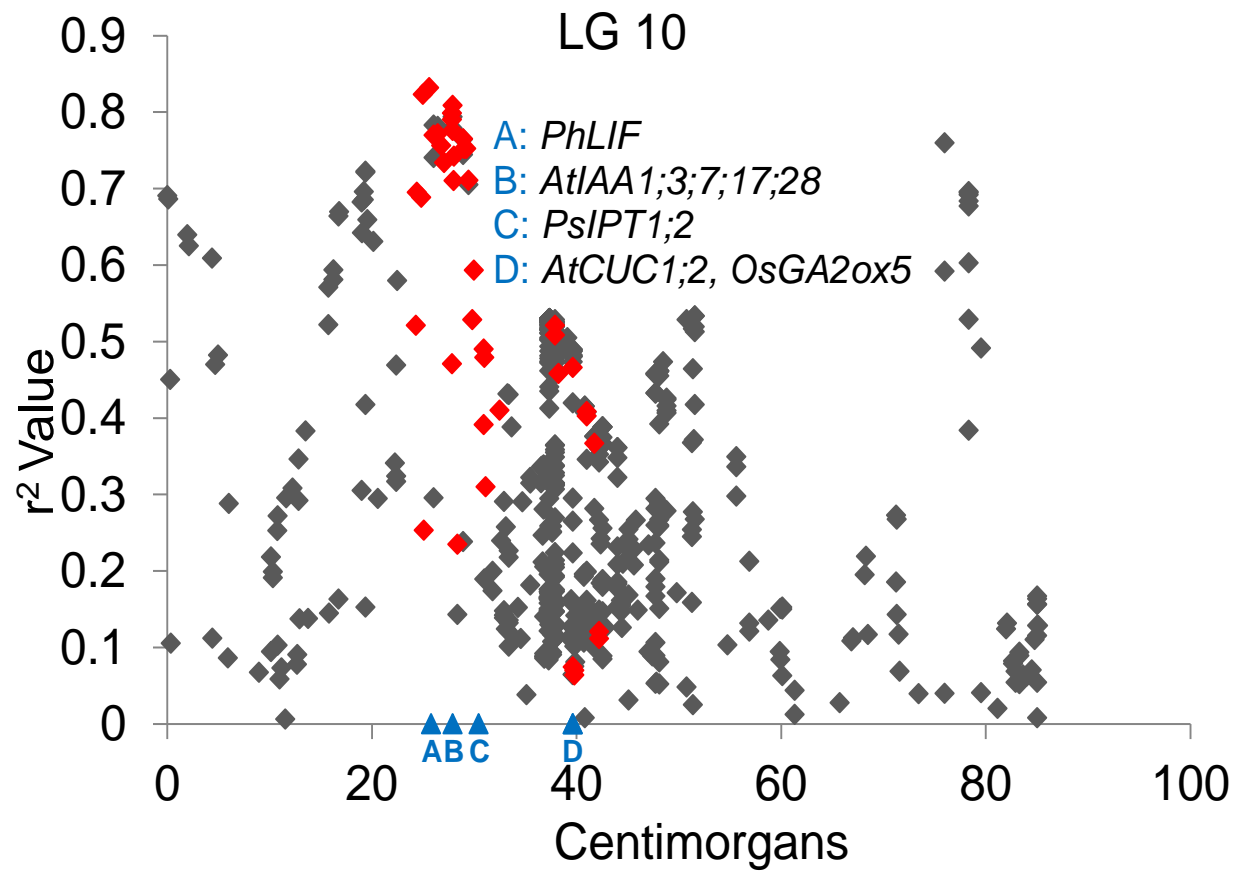

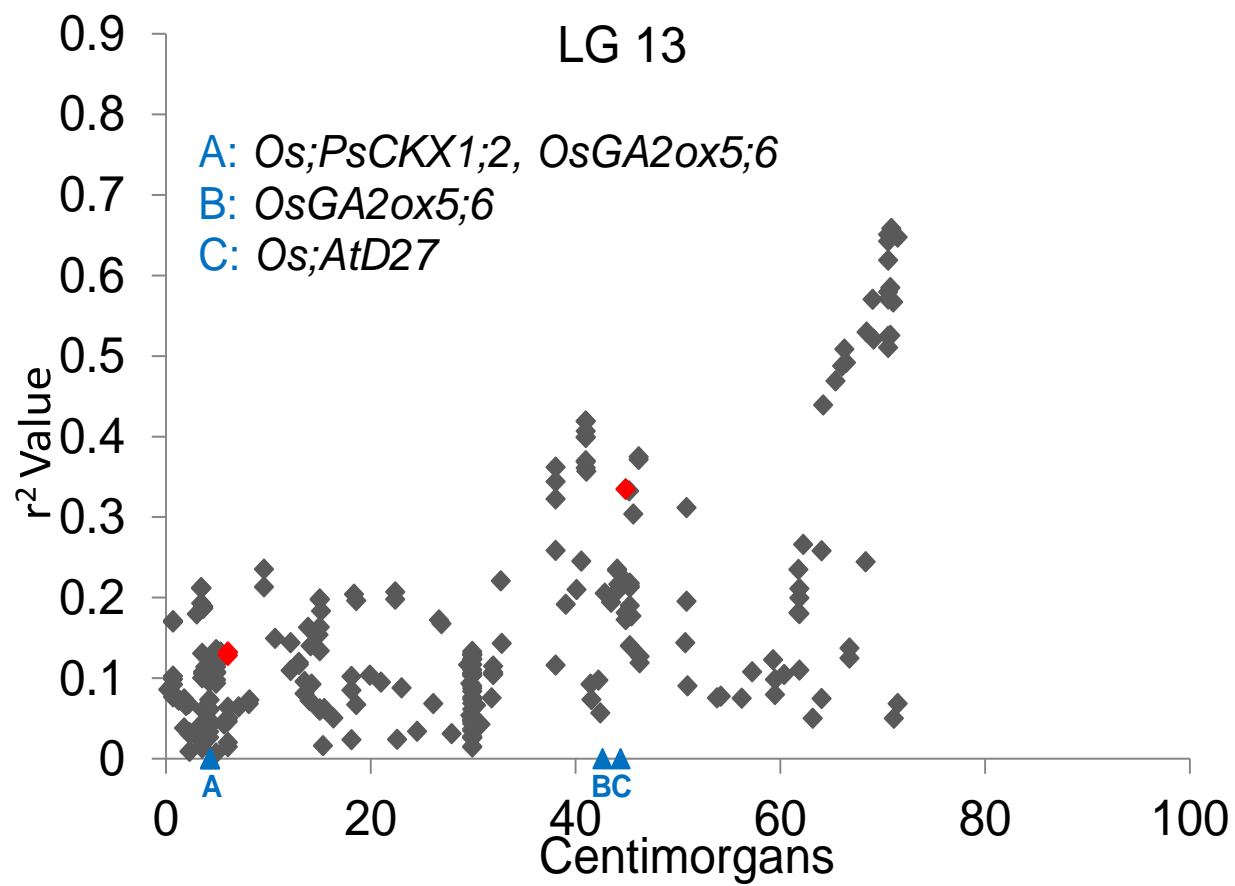

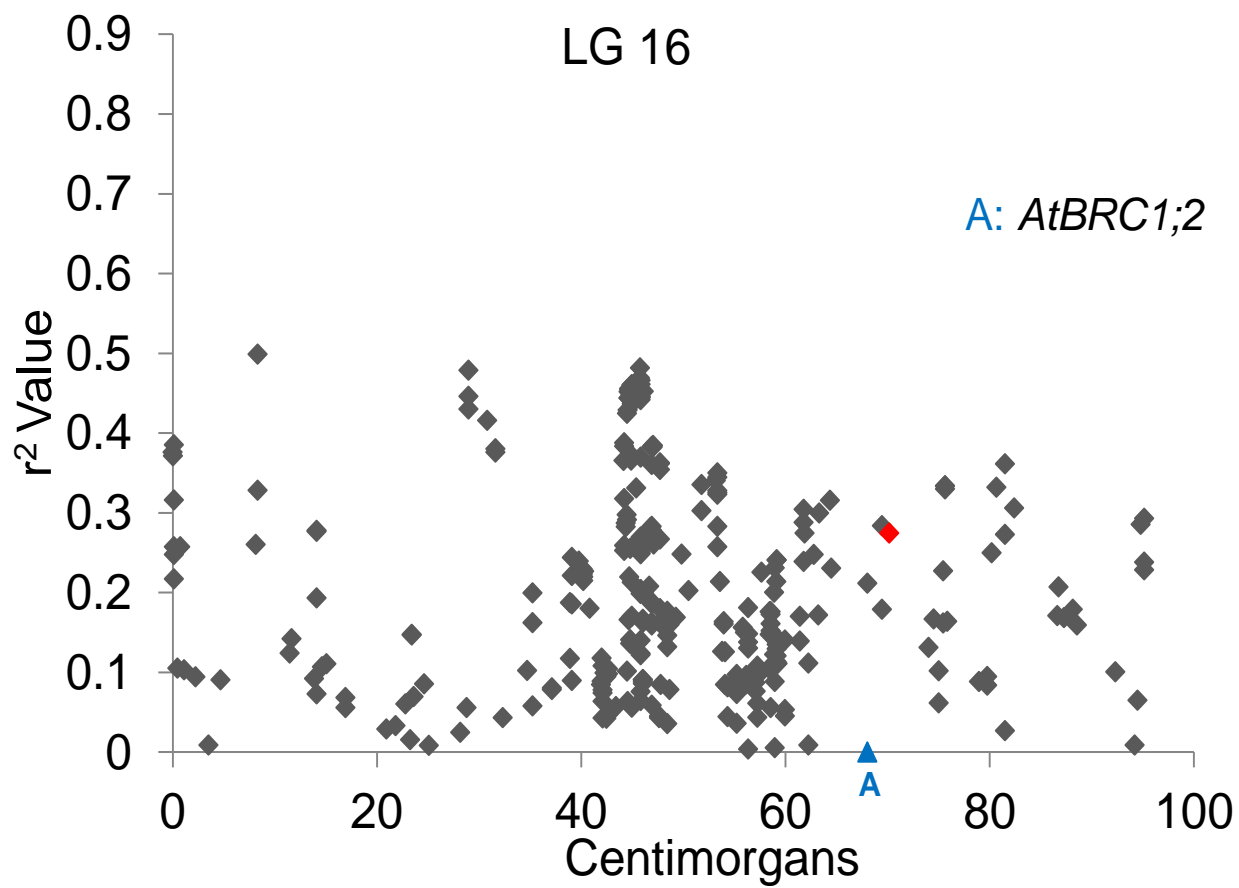

Supplement: Additional file 6: — Average r 2 values for SNPs along LGs that contained candidate branching genes that co-localized with significant branching associations. Average r 2 values were calculated as described in Figure 6. SNPs exhibiting significant branching associations and located in close proximity to a candidate branching gene are depicted in red. The positions of the putative candidate genes are indicated by blue arrows on the x-axis; letters refer to specific genes. [file 12870_2015_458_MOESM6_ESM.pdf]
