# Supplementary material for: Association mapping in sunflower (Helianthus annuus L.) reveals independent control of apical vs. basal branching
Source: BMC Plant Biol. 2015 Mar 11;15:84. doi: 10.1186/s12870-015-0458-9 (PMC4407831; doi:10.1186/s12870-015-0458-9)
Supplement: Additional file 5: — Sunflower genetic map depicting the positions of significant SNPs associated with branching traits and candidate branching genes. Shown are all 17 LGs and positions of all significant SNPs found in the study. Candidate branching genes are in black. Branching traits associated with the SNPs are color coded as in Figure 5 with apical (A; in green), mid-apical (MA; in dark blue), mid (M; in orange), mid-basal (MB; in magenta), basal (B; in brown), and secondary branching (2B; in light blue). Locations are indicated as follows: G=Georgia, I=Iowa, and C=British Columbia). For LG 10 only, SNPs associated with all branching traits other than 2B are indicated as whole plant branching (WPB) and the numbers denote differences among geographical locations for a given branching type. [file 12870_2015_458_MOESM5_ESM.pdf]

Additional file 4

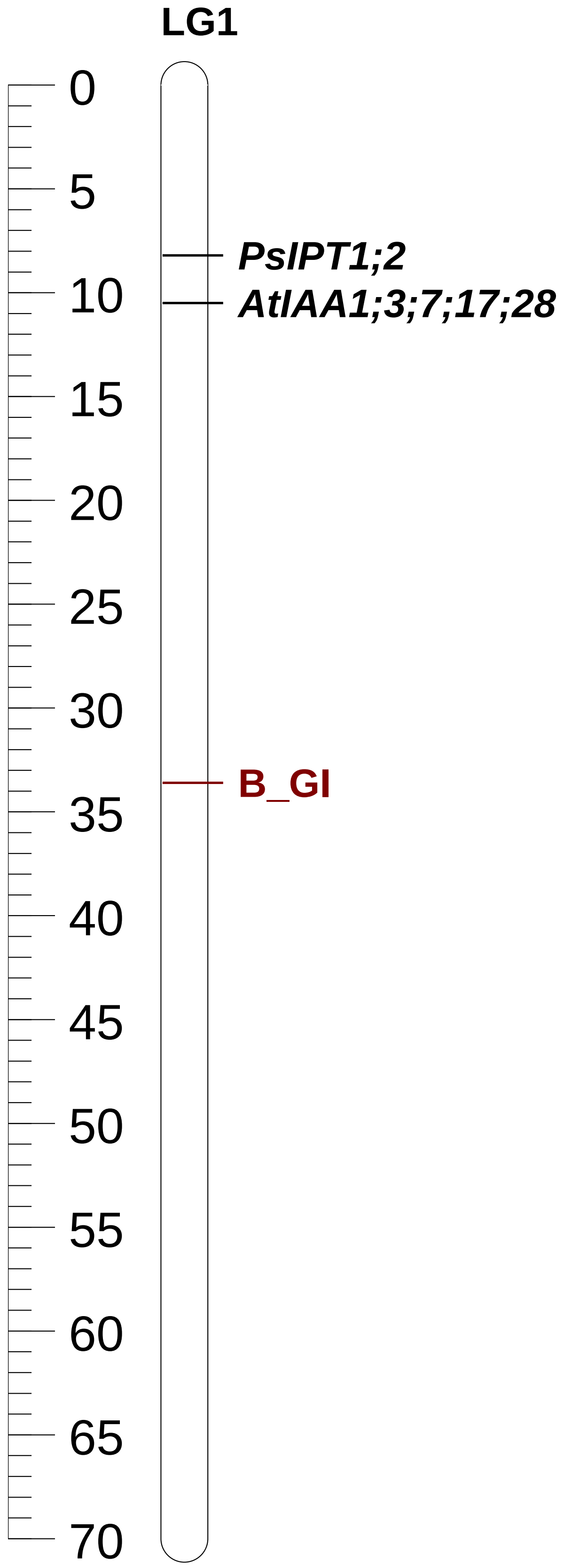

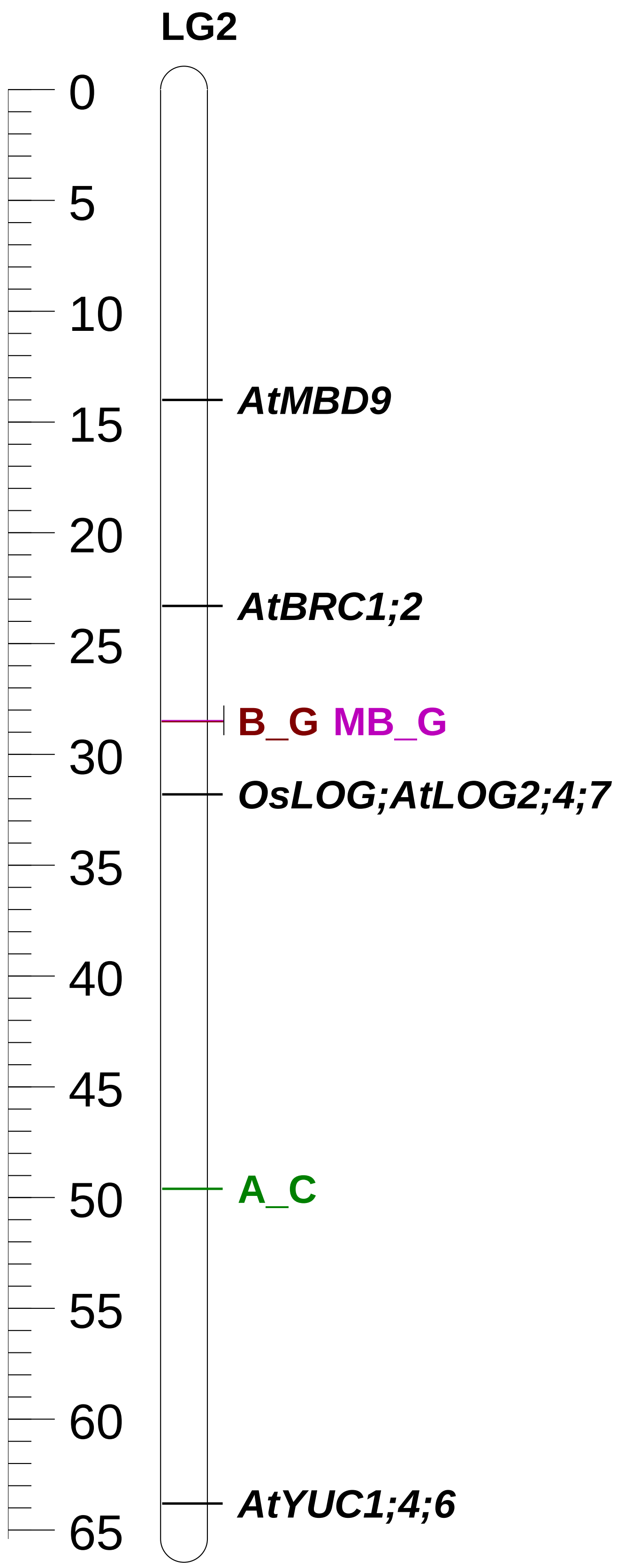
LG2
0
5
10
15
20
25
30
35
40
45
50
55
60
65
AtMBD9
AtBRC1;2
B_G MB_G
OsLOG;AtLOG2;4;7
A_C
AtYUC1;4;6

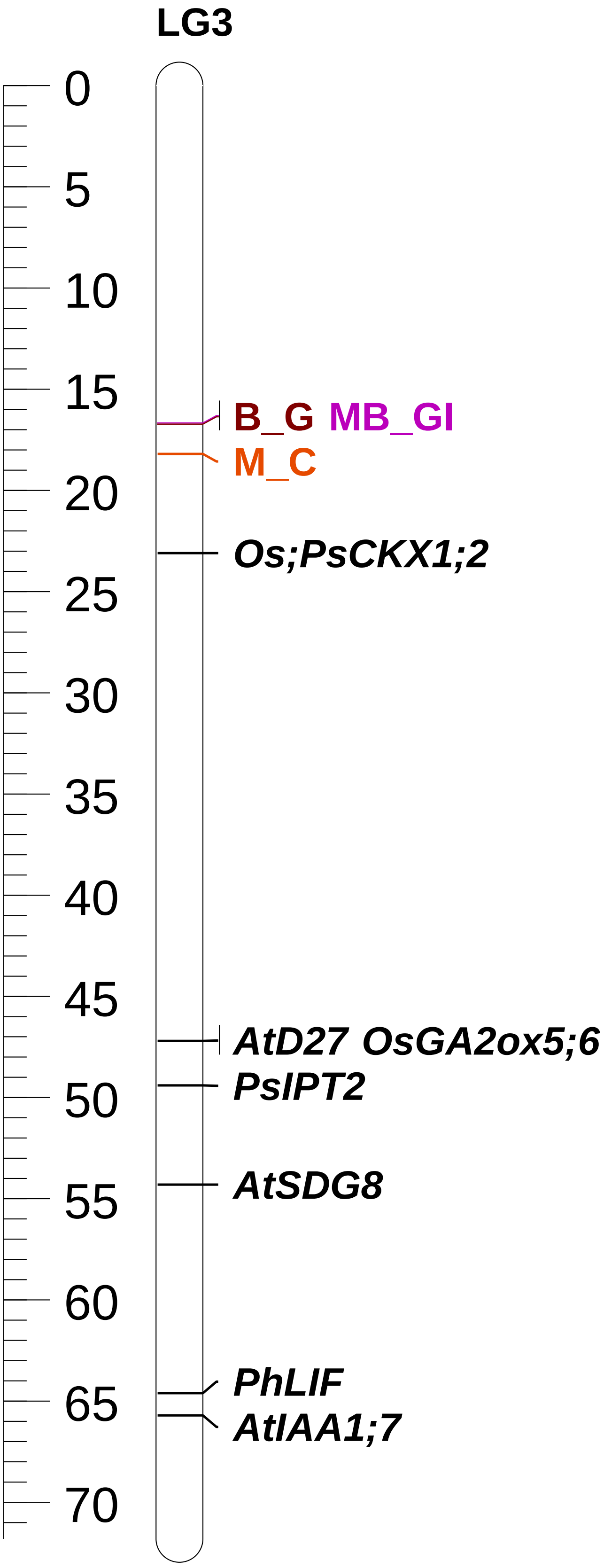
LG3
0
5
10
15
20
25
30
35
40
45
50
55
60
65
70
B_G MB_GI
M_C
Os;PsCKX1;2
AtD27 OsGA2ox5;6
PsIPT2
AtSDG8
PhLIF
AtIAA1;7

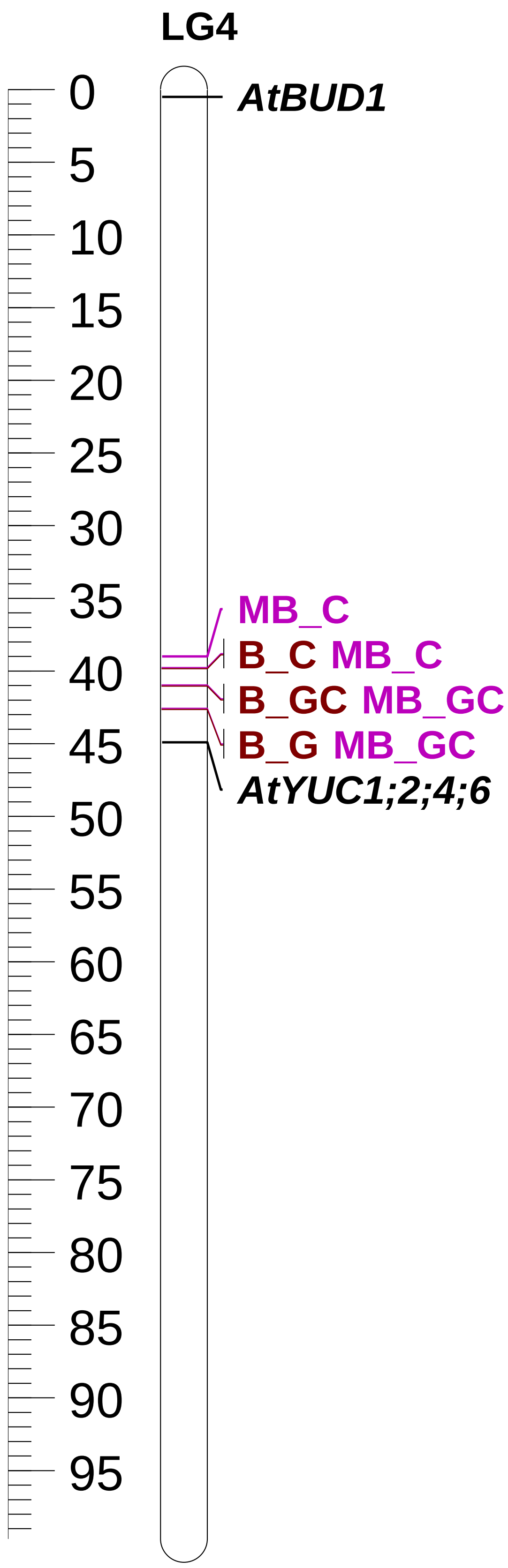

LG4
0
5
10
15
20
25
30
35
40
45
50
55
60
65
70
75
80
85
90
95
AtBUD1
MB_C
B_C MB_C
B_GC MB_GC
B_G MB_GC
AtYUC1;2;4;6

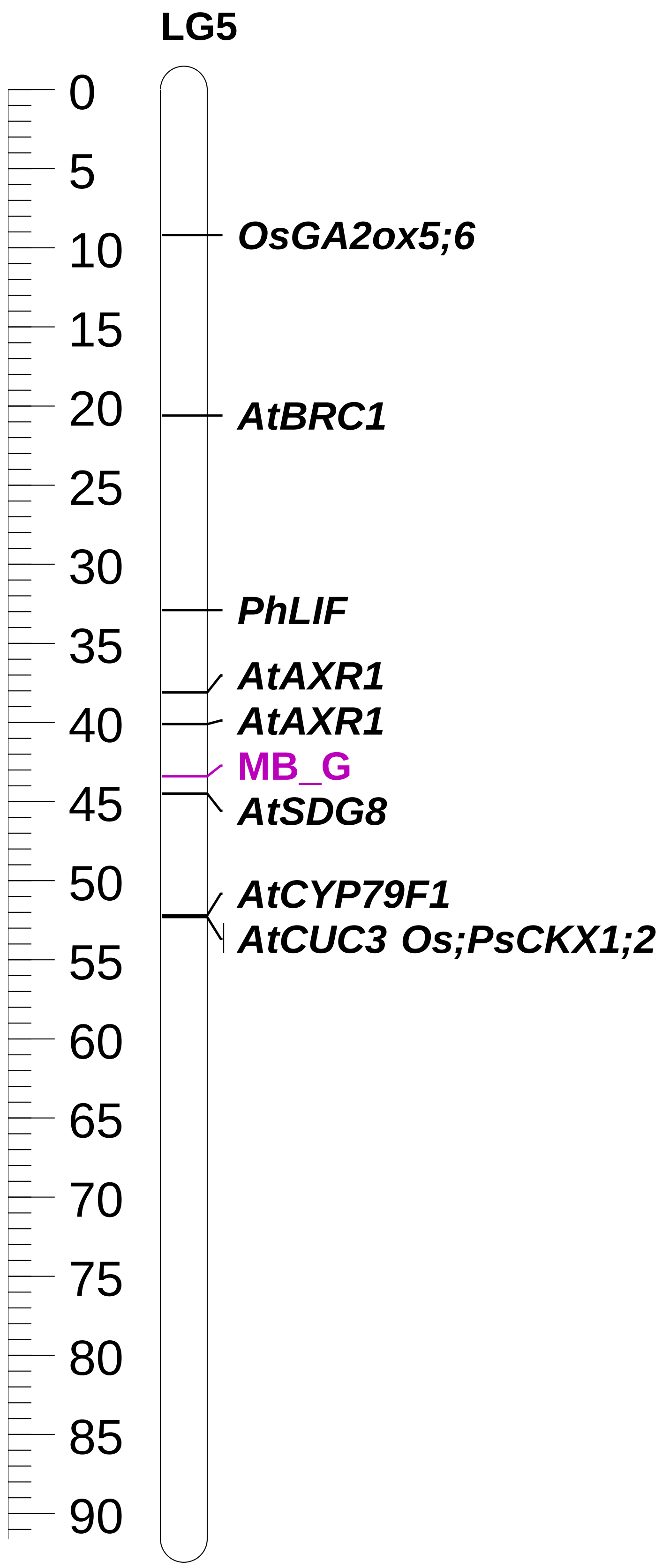
LG5
0
5
10
15
20
25
30
35
40
45
50
55
60
65
70
75
80
85
90
OsGA2ox5;6
AtBRC1
PhLIF
AtAXR1
AtAXR1
MB_G
AtSDG8
AtCYP79F1
AtCUC3 Os;PsCKX1;2

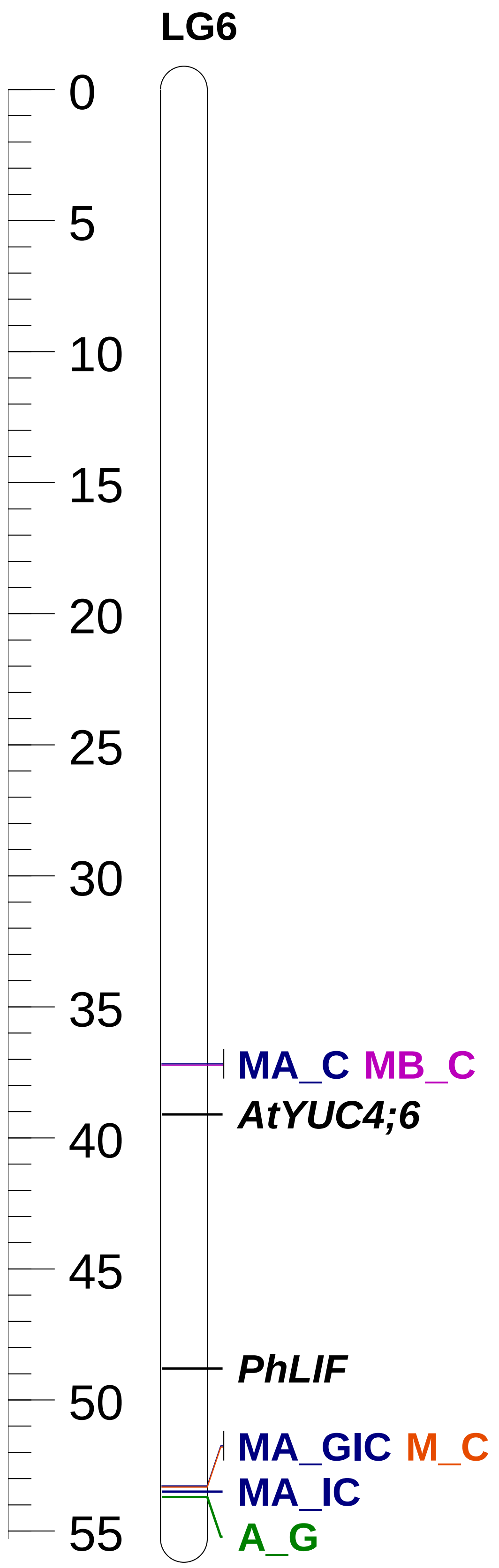
LG6
0
5
10
15
20
25
30
35
40
45
50
55
MA_C MB_C
AtYUC4;6
PhLIF
MA_GIC M_C
MA_IC
A_G

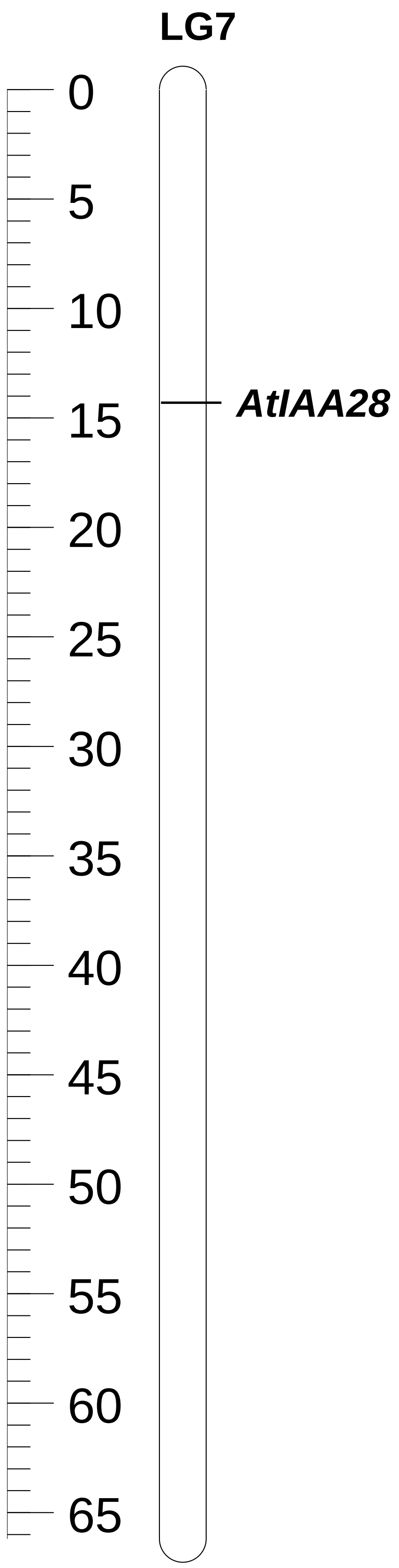
LG7
0
5
10
15
20
25
30
35
40
45
50
55
60
65
AtIAA28

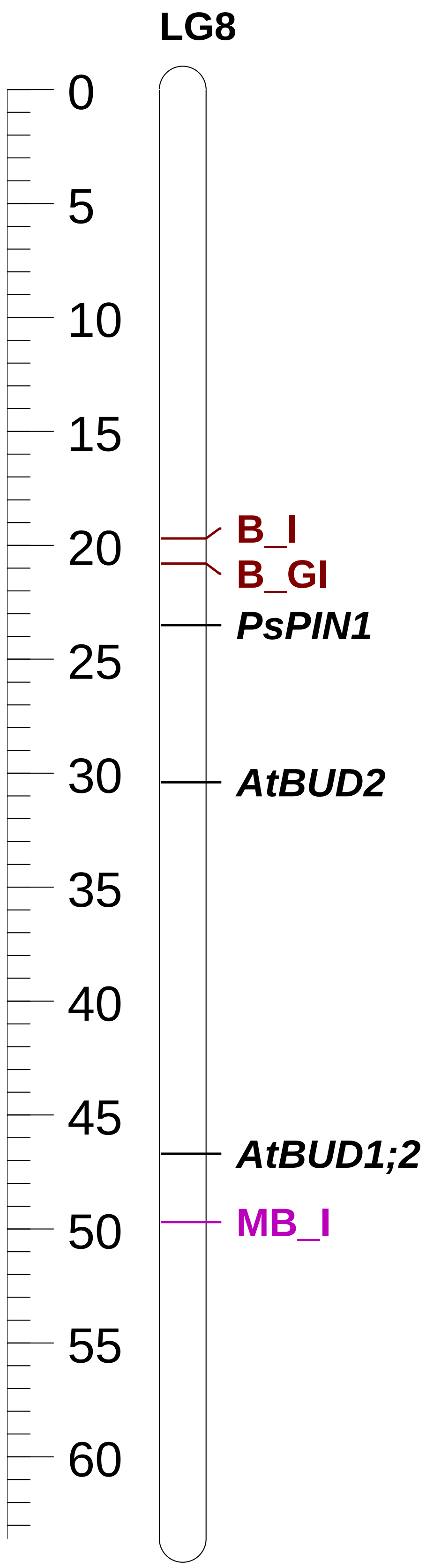
LG8
0
5
10
15
20
25
30
35
40
45
50
55
60
B_I
B_GI
PsPIN1
AtBUD2
AtBUD1;2
MB_I

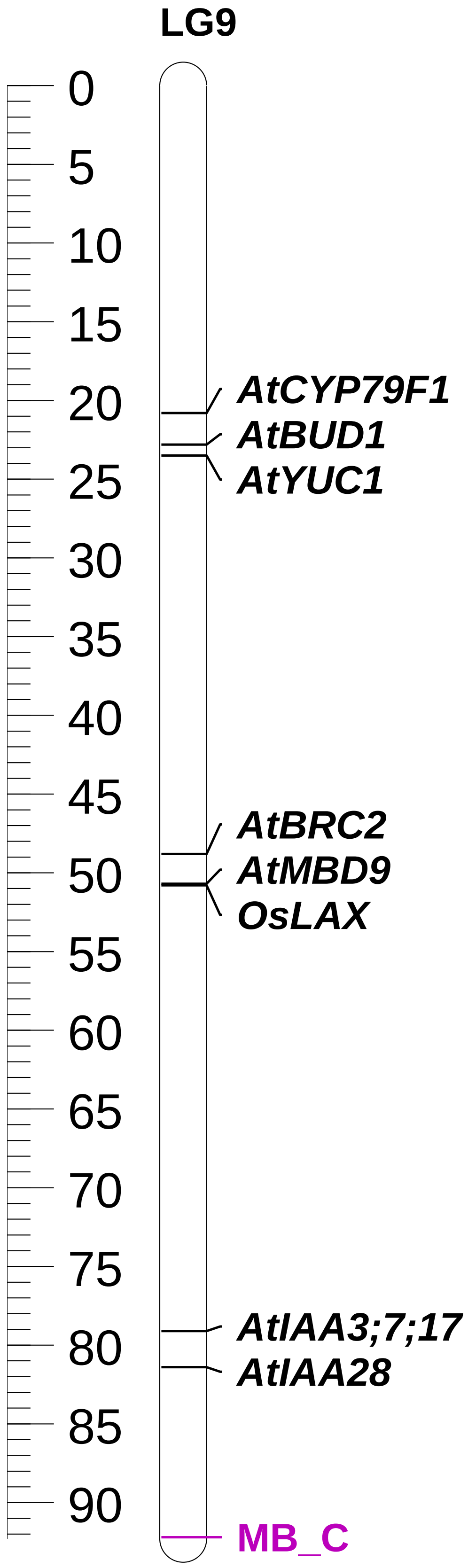
LG9
0
5
10
15
20
25
30
35
40
45
50
55
60
65
70
75
80
85
90
AtCYP79F1
AtBUD1
AtYUC1
AtBRC2
AtMBD9
OsLAX
AtIAA3;7;17
AtIAA28
MB_C

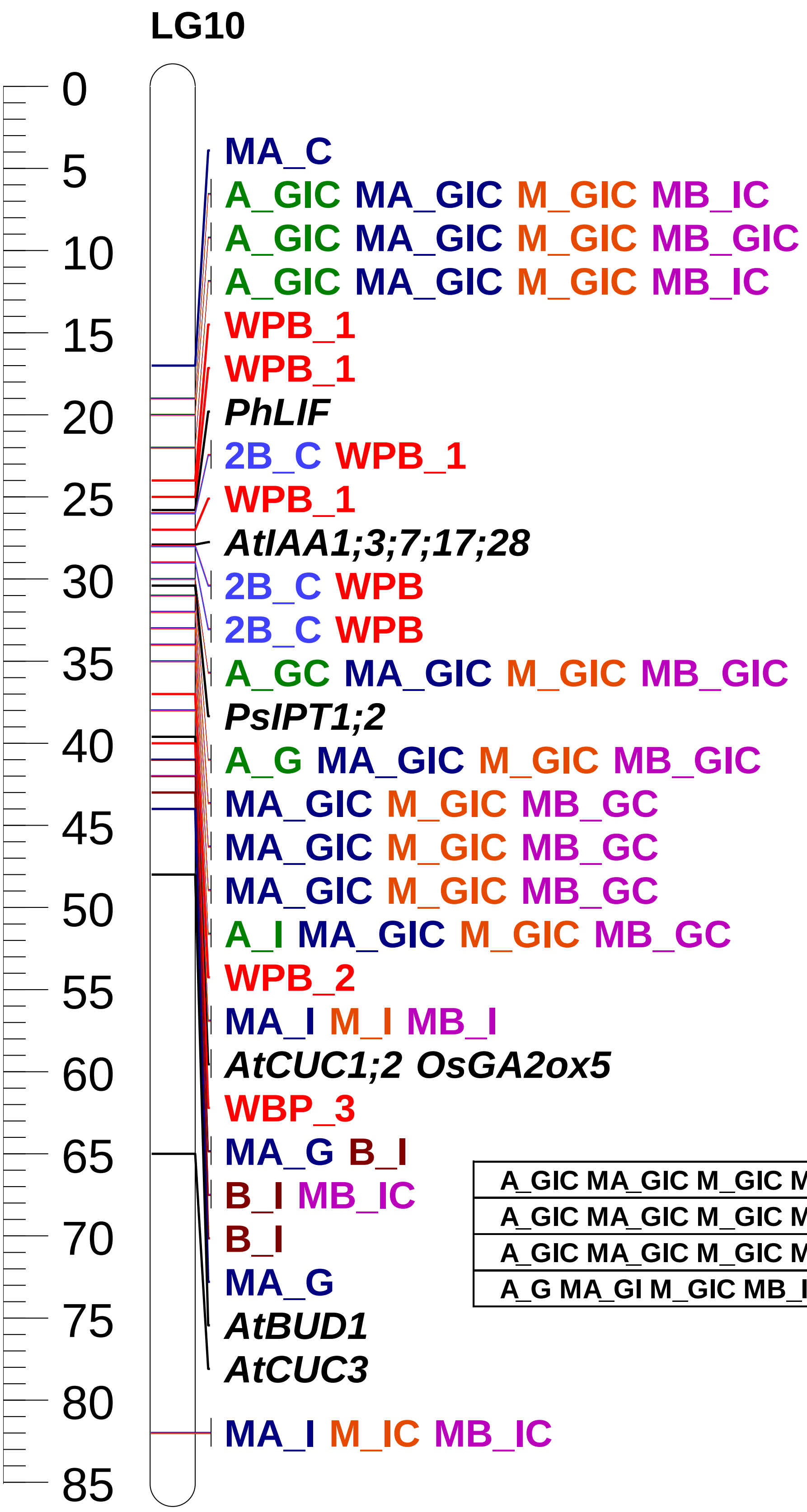

A_GIC MA_GIC M_GIC MB_GIC B_IC	WPB
A_GIC MA_GIC M_GIC MB_GIC B_C	WPB_1
A_GIC MA_GIC M_GIC MB_GIC B_I	WPB_2
A_G MA_GI M_GIC MB_IC B_IC	WPB_3

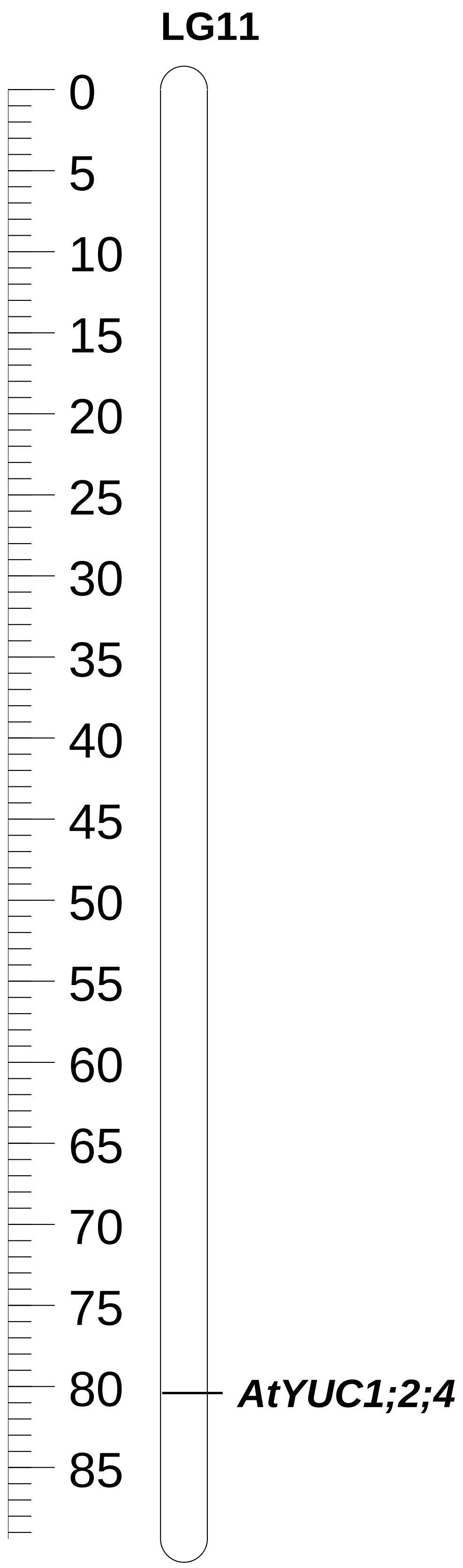

LG11
0
5
10
15
20
25
30
35
40
45
50
55
60
65
70
75
80
85
AtYUC1;2;4

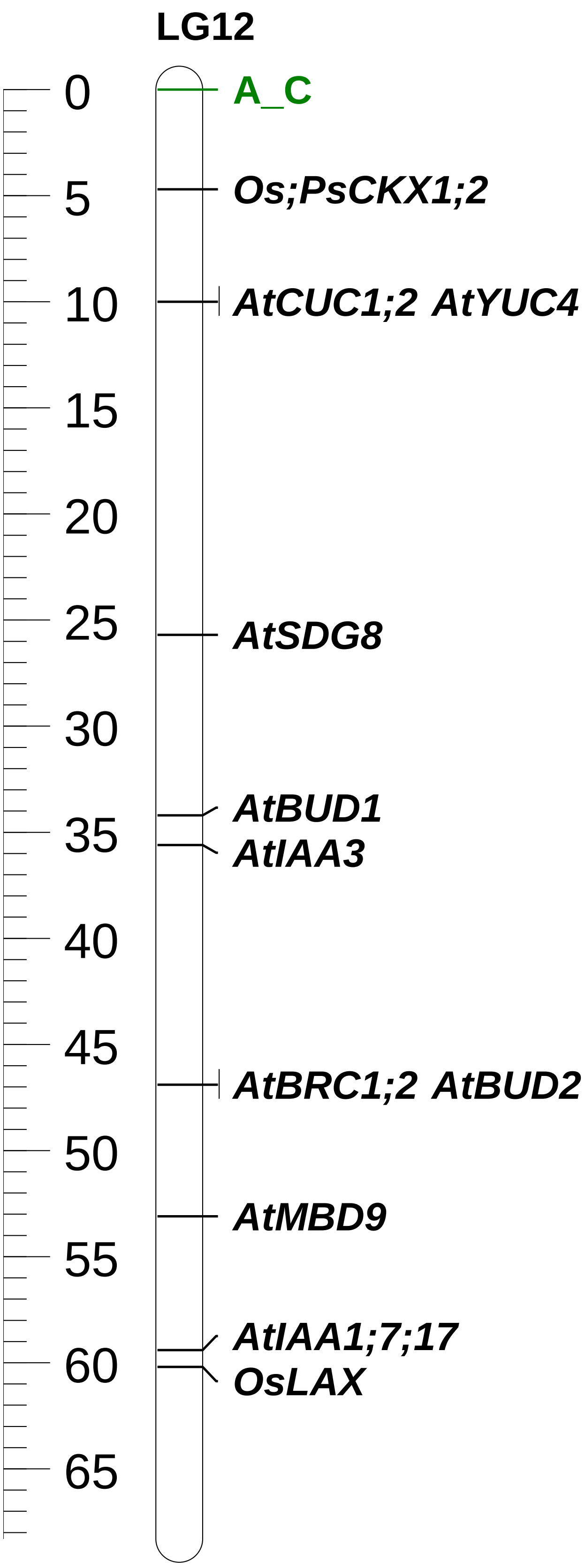
LG12
0
5
10
15
20
25
30
35
40
45
50
55
60
65
A_C
Os;PsCKX1;2
AtCUC1;2 AtYUC4
AtSDG8
AtBUD1
AtIAA3
AtBRC1;2 AtBUD2
AtMBD9
AtIAA1;7;17
OsLAX

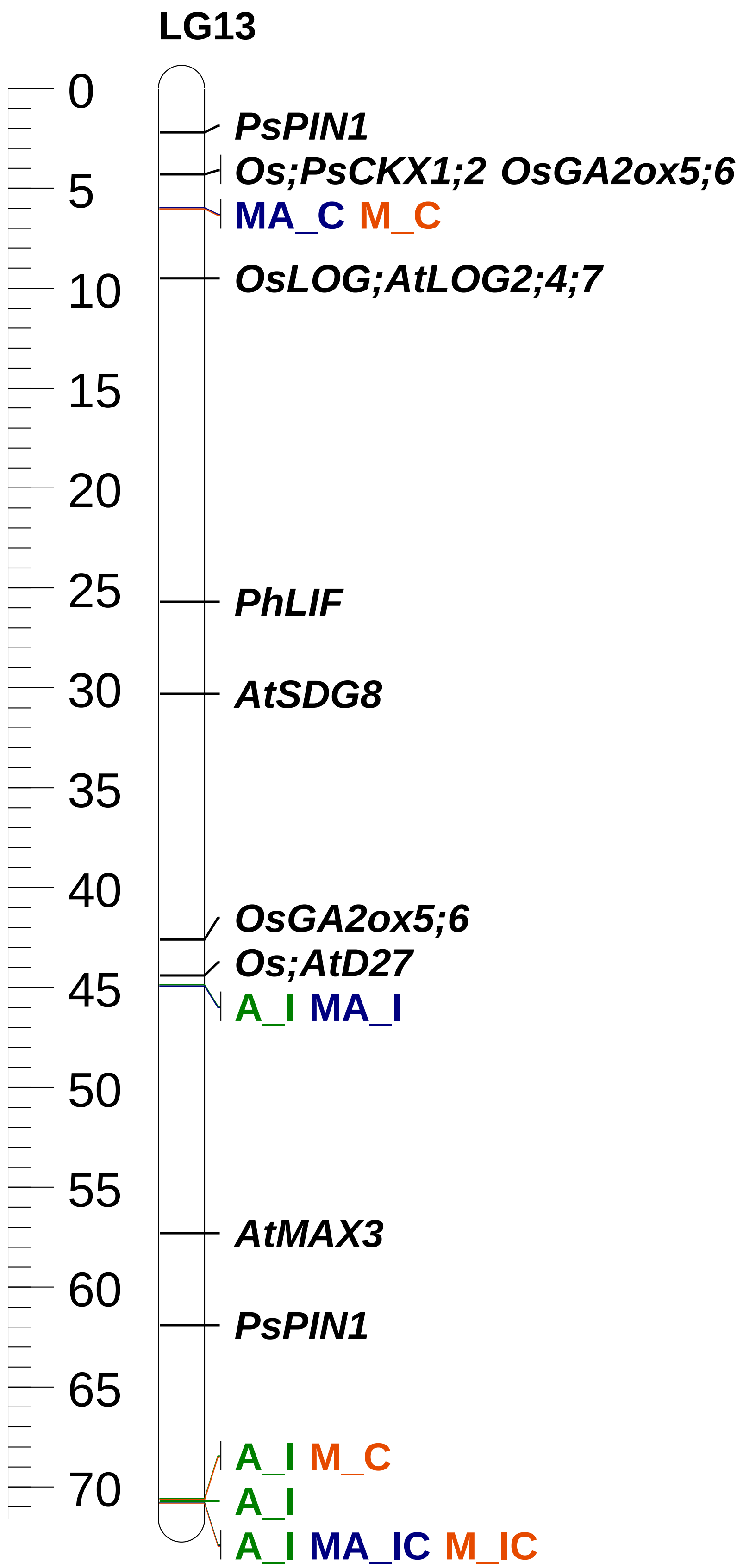
LG13
0
5
10
15
20
25
30
35
40
45
50
55
60
65
70
PsPIN1
Os;PsCKX1;2 OsGA2ox5;6
MA_C M_C
OsLOG;AtLOG2;4;7
PhLIF
AtSDG8
OsGA2ox5;6
Os;AtD27
A_I MA_I
AtMAX3
PsPIN1
A_I M_C
A_I
A_I MA_IC M_IC

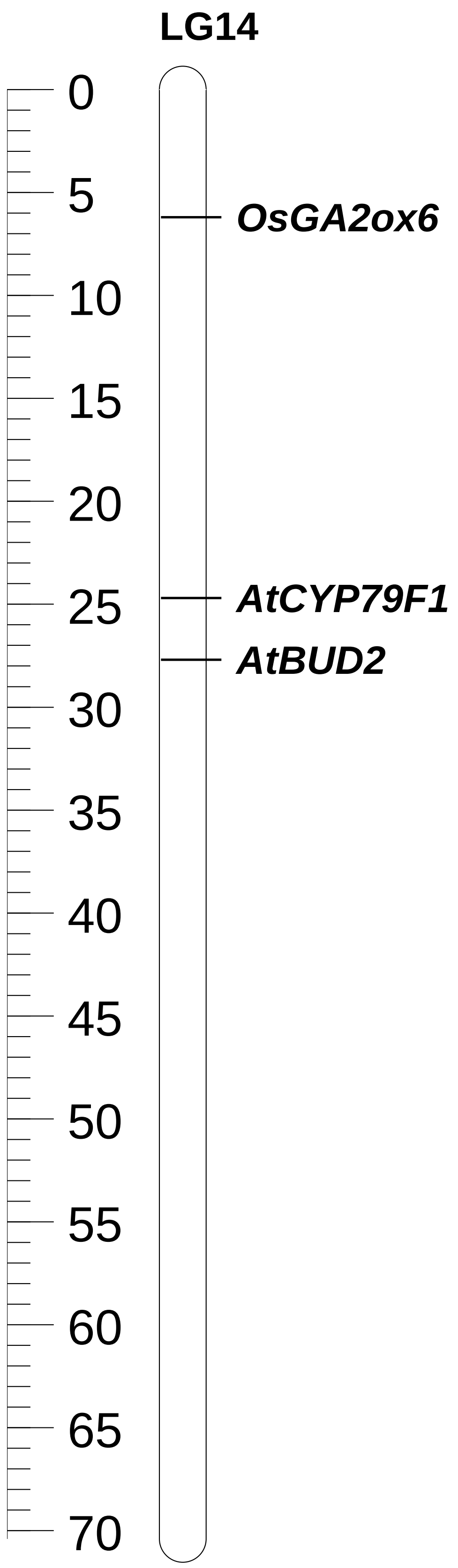

LG14
0
5
10
15
20
25
30
35
40
45
50
55
60
65
70
OsGA2ox6
AtCYP79F1
AtBUD2

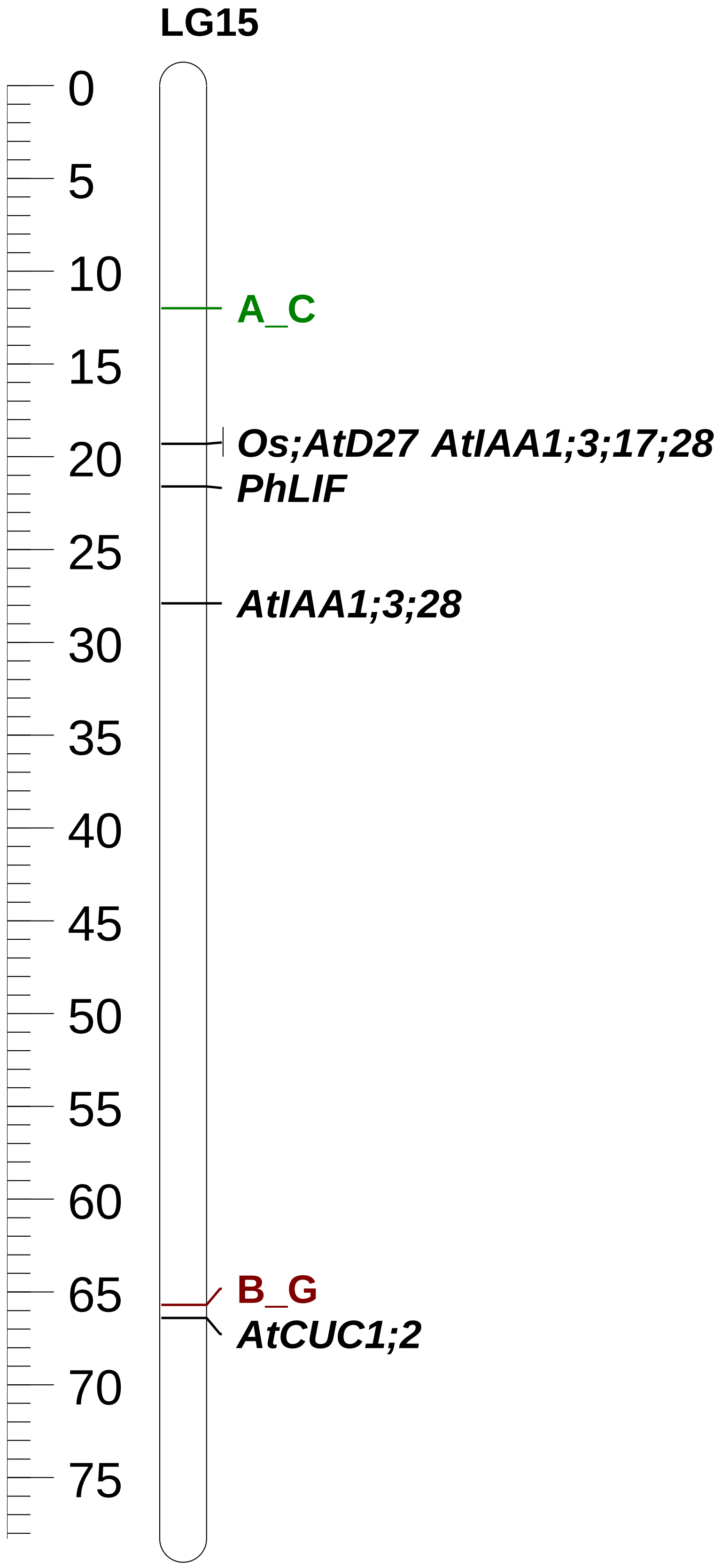

LG15
0
5
10
15
20
25
30
35
40
45
50
55
60
65
70
75
A_C
Os;AtD27 AtIAA1;3;17;28
PhLIF
AtIAA1;3;28
B_G
AtCUC1;2

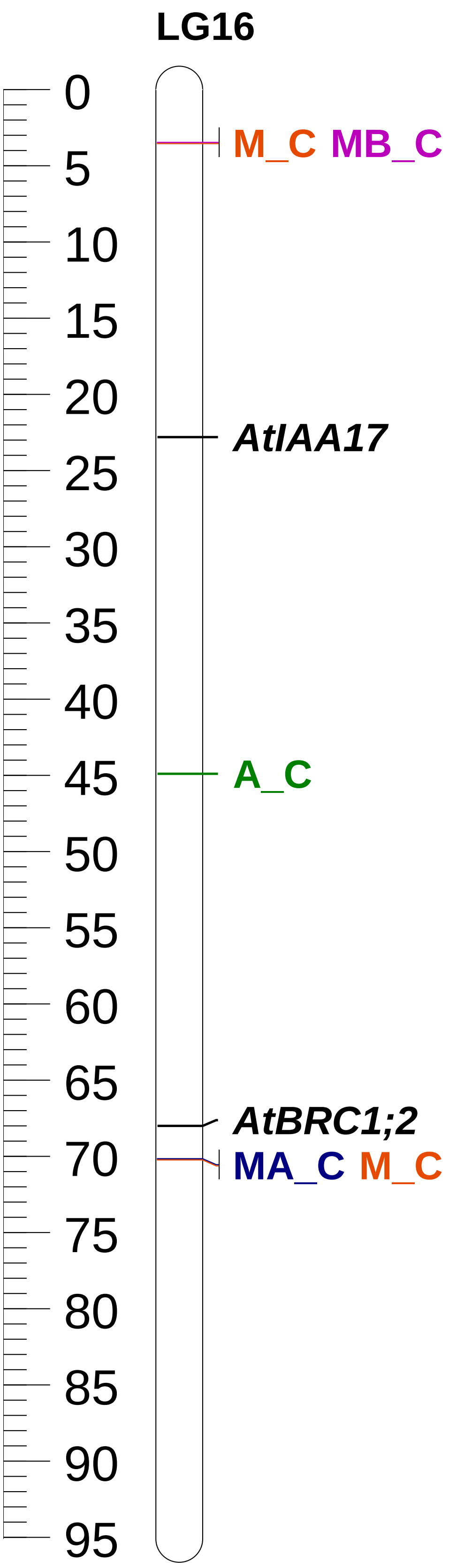

LG16
0
5
10
15
20
25
30
35
40
45
50
55
60
65
70
75
80
85
90
95
M_C MB_C
AtIAA17
A_C
AtBRC1;2
MA_C M_C

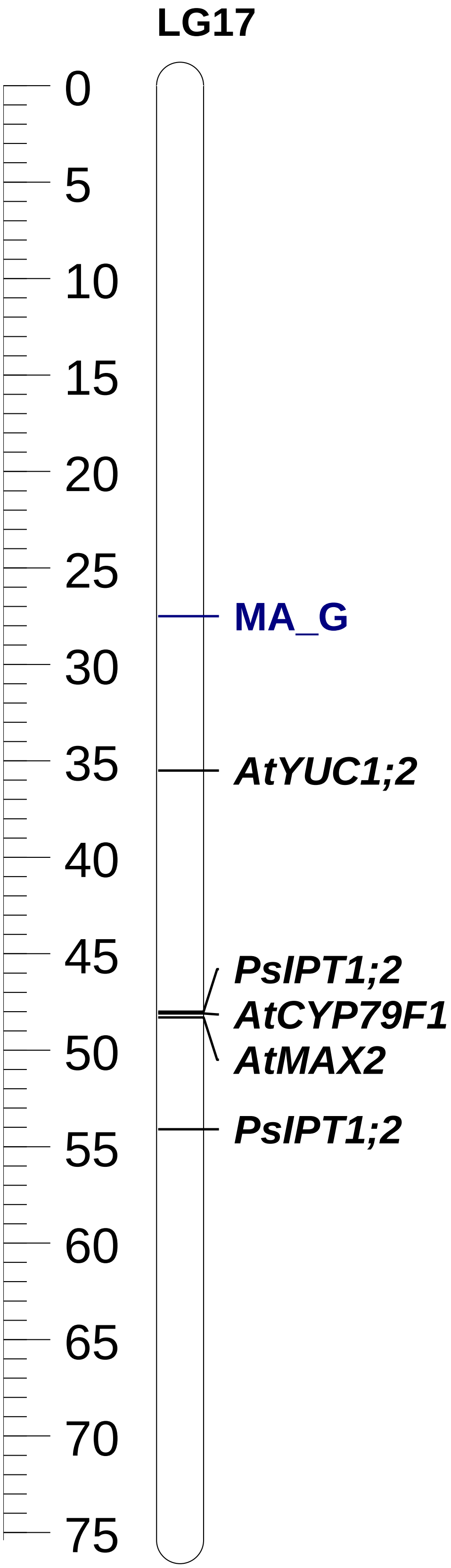
LG17
0
5
10
15
20
25
30
35
40
45
50
55
60
65
70
75
MA_G
AtYUC1;2
PsIPT1;2
AtCYP79F1
AtMAX2
PsIPT1;2
